# Premature Senescence and Increased TGFβ Signaling in the Absence of Tgif1

**DOI:** 10.1371/journal.pone.0035460

**Published:** 2012-04-13

**Authors:** Brad J. Zerlanko, Laurent Bartholin, Tiffany A. Melhuish, David Wotton

**Affiliations:** Department of Biochemistry and Molecular Genetics and Center for Cell Signaling, University of Virginia, Charlottesville, Virginia, United States of America; Chinese University of Hong Kong, Hong Kong

## Abstract

Transforming growth factor β (TGFβ) signaling regulates cell cycle progression in several cell types, primarily by inducing a G1 cell cycle arrest. Tgif1 is a transcriptional corepressor that limits TGFβ responsive gene expression. Here we demonstrate that primary mouse embryo fibroblasts (MEFs) lacking Tgif1 proliferate slowly, accumulate increased levels of DNA damage, and senesce prematurely. We also provide evidence that the effects of loss of Tgif1 on proliferation and senescence are not limited to primary cells. The increased DNA damage in *Tgif1* null MEFs can be partially reversed by culturing cells at physiological oxygen levels, and growth in normoxic conditions also partially rescues the proliferation defect, suggesting that in the absence of Tgif1 primary MEFs are less able to cope with elevated levels of oxidative stress. Additionally, we show that *Tgif1* null MEFs are more sensitive to TGFβ-mediated growth inhibition, and that treatment with a TGFβ receptor kinase inhibitor increases proliferation of *Tgif1* null MEFs. Conversely, persistent treatment of wild type cells with low levels of TGFβ slows proliferation and induces senescence, suggesting that TGFβ signaling also contributes to cellular senescence. We suggest that in the absence of Tgif1, a persistent increase in TGFβ responsive transcription and a reduced ability to deal with hyperoxic stress result in premature senescence in primary MEFs.

## Introduction

In response to transforming growth factor (TGF) β signaling Smad2 and Smad3 are phosphorylated by TGFβ type I receptors, associate with Smad4 and accumulate in the nucleus, where they activate target gene expression [1]–[3]. TGFβ signaling has antiproliferative effects in several cell types, including epithelial cells and primary MEFs [4]. TGFβ induces cell cycle arrest, in part, by increasing expression of CDK inhibitors, such as p15 and p21, and by decreasing expression of growth promoters, such as c-Myc [5]–[7]. The cytostatic effects of TGFβ generally result in a G1 arrest, and loss of this growth inhibitory effect due to inactivation of components of the TGFβ pathway is associated with tumorigenesis [8], [9].

Tgif1 (thymine guanine interacting factor) is a homeodomain protein of the TALE (three amino acid loop extension) superfamily [10], [11]. Tgif family members are characterized by the highly conserved homeodomain and a carboxyl-terminal extension [12]. Loss of function mutations in human *TGIF1*, have been linked to holoprosencephaly, which is a devastating developmental disease affecting craniofacial development [13], [14]. Several groups have created *Tgif1* null mutations in mice, without any strong phenotypes on a mixed strain background [15]–[18]. On a C57BL/6 strain background complete loss of Tgif1 results in placental defects and some perinatal lethality [19]. A null mutation in mouse *Tgif2* does not cause significant phenotypes on a mixed strain background. However, loss of both Tgif1 and Tgif2 together causes gastrulation defects and embryonic lethality, clearly suggesting essential overlapping functions, at least during early embryogenesis [20]. In embryos lacking both Tgif1 and Tgif2, the gastrulation defects could be partially rescued by genetically reducing the dose of Nodal, supporting an *in vivo* role for Tgifs in the Nodal/TGFβ signaling pathway [20].

Activated Smad complexes can bind directly to DNA, or can be recruited indirectly via other DNA binding proteins, and then activate transcription via interactions with general coactivators [2]. Tgifs interact with Smad2 and Smad3 in response to TGFβ signaling, and repress Smad target gene expression [21], [22]. The interaction of Tgifs with Smad2/3 results in displacement of coactivators and the recruitment of transcriptional corepressors, thereby limiting transcriptional activation in response to TGFβ. Tgif1 and Tgif2 interact with mSin3A via a conserved repression domain close to their carboxyl-termini [23], [24]. In addition, Tgif1 contains an amino-terminal PLDLS motif that recruits the CtBP1 and CtBP2 corepressors [25]. The DNA binding site for Tgifs is known, and human Tgif1 was first identified by its ability to bind to a consensus motif adjacent to a retinoid X receptor (RXR) binding sequence from the rat *Crbp2* gene [10]. Binding of TGIF1 to this element reduced transcriptional activation by RXR. More recently, TGIF1 has been shown to bind to the RXR, suggesting that it may be a more general repressor of retinoid signaling [15]. Since RXR is a common heterodimeric partner of many nuclear receptors (NR) Tgifs might repress other NR transcriptional responses, and there is evidence that RXR-LXR heterodimers are preferential targets for Tgif1 in mouse liver [26]. Thus Tgifs may regulate pathways in addition to those activated by TGFβ signals.

Mouse embryo fibroblasts (MEFs) are primary cells with limited life-span, that senesce in culture [27], [28]. Mutations in a number of genes encoding transcriptional regulators, including Sirt6 and c-Jun, exacerbate the senescent phenotype in primary MEFs [29], [30]. With increasing passage number, primary wild type MEFs proliferate more slowly and the cells take on a flatter more spread out appearance that is characteristic of senescence. At later passages senescence associated β-galactosidase (SAβG) activity can be detected, and a larger proportion of the cells become tetraploid and arrest with 8N DNA content [31]. Deletion of p53 weakens the spindle checkpoint and accelerates the rate at which MEFs become tetraploid [31], [32]. Deletion of the three Rb-related pocket proteins (Rb, p107 and p130) prevents senescence and results in immortalization, consistent with disruption of a tetraploidy checkpoint [33]. However, there is evidence suggesting that tetraploidy alone does not trigger checkpoint mediated growth arrest [34]. The senescence observed in primary MEFs is thought to be due at least in part to the stress of being placed in culture [28]. A major stress of tissue culture is growth under hyperoxic conditions, which results in accumulation of DNA damage [27]. Growth in more physiological oxygen levels decreases DNA damage in MEFs. For example, primary MEFs lacking c-Jun have increased DNA damage, increased ploidy, and undergo premature senescence [29]. Growth in reduced oxygen reverses the DNA damage phenotype and delays the onset of senescence.

We demonstrate here that primary MEFs lacking *Tgif1* have proliferation defects and early senescence. *Tgif1* null cells are more sensitive to hyperoxic stress and have higher levels of DNA damage than wild type cells. Additionally, at early passage TGFβ signaling contributes to the reduced proliferation in *Tgif1* null cells. Persistent low level TGFβ stimulation of wild type MEFs results in decreased proliferation and an increase in senescence. Thus we provide evidence that a combination of increased DNA damage and increased activity of the TGFβ pathway contribute to the proliferation defects observed in the absence of Tgif1, suggesting two independent pathways that contribute to senescence.

## Results

### Decreased proliferation and premature senescence in *Tgif1* null MEFs

**Figure 1:**
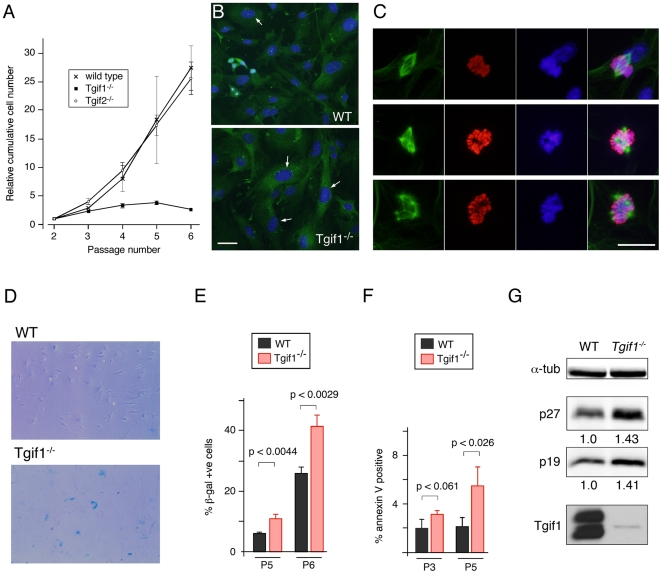
A proliferation defect in *Tgif1* null MEFs. A) Triplicate cultures of primary MEFs of the indicated genotypes were grown on a 3T3 protocol. Relative cumulative cell number is plotted against passage number. The starting number of cells (300,000) plated at passage 2 was set equal to 1 for each genotype. B) Wild type and *Tgif1* null passage 5 MEFs were examined by indirect immunofluorescence with an antibody against γ-tubulin (green) to identify centrosomes, and co-stained with DAPI for DNA (blue). Images were captured at 20×. Arrows indicate nuclei of cells with more than 2 centrosomes. Scale bar = 50 µM. C) Representative images of passage 3 cells analyzed for α-tubulin (green), pHH3 (red), and Hoechst (blue) are shown, together with a merged image of all three colors. The upper panels show a normal bipolar mitosis in a wild type cell. The lower panels show examples of multi-polar mitoses in *Tgif1* null and wild type cells. Images were captured at 20×. Scale bar = 25 µM. D) Passage 5 and 6 wild type and *Tgif1* null MEFs were stained with X-gal to detect endogenous β-galactosidase activity. E) The percentage of β-galactosidase positive cells was quantified for wild type and *Tgif1* null MEFs at passages 5 and 6, from triplicate cultures. F) Passage 3 and 5 cells were stained with annexin V to detect apoptotic cells. The proportion of positive cells (average plus s.d. of triplicate cultures) is shown. Significance as determined by Student's T test is shown. G) Passage 2 wild type and *Tgif1* null MEFs were analyzed by western blotting for the CDK inhibitors, p27 and p19, and for α-tubulin and Tgif1 as controls. The relative expression of p27 and p19 (normalized to α-tubulin) is shown below each blot.

When we attempted to culture MEFs isolated from mice lacking *Tgif1*, we found that they grew poorly relative to wild type cells. Additionally, a proliferative defect was reported in *Tgif1* null MEFs generated by a different targeting strategy [17]. As an initial characterization of the ability to proliferate we performed 3T3 assays on wild type, *Tgif1* null and *Tgif2* null MEFs. Wild type and *Tgif2* null MEFs proliferated robustly over the first six passages, whereas, *Tgif1* null MEFs proliferated significantly less well even at passages 3 and 4 (Figure 1A). We also determined the proportion of cells in S phase: Cells at passages 4 to 6 were incubated with EdU for 1 hour, and observed by fluorescence microscopy. The proportion of cells incorporating EdU decreased with passage number and was lower in *Tgif1* null MEFs than in wild type cells (data not shown). We next used an antibody against phosphorylated histone H3 (pHH3) to identify cells in late G2 or mitosis. As with EdU labeling, the proportion of G2/M cells decreased at later passages with fewer present in the *Tgif1* null culture (data not shown). While analyzing MEFs by microscopy, we noticed that *Tgif1* null cells appeared to be larger and flatter with larger nuclei than the wild type MEFs. When we stained *Tgif1* null MEFs with an antibody against γ-tubulin many of these cells had more than two γ-tubulin foci, consistent with having multiple centrosomes (Figure 1B). To determine whether *Tgif1* null MEFs had multiple spindles, we stained cells for pHH3 to identify cells in late G2 and mitosis, α-tubulin to identify microtubules, and with Hoechst for DNA. Based on the pHH3 and Hoechst stain, we identified cells that were in late mitosis and which, based on the α-tubulin staining, had clearly formed mitotic spindles. In most cases, where spindles were evident, the cells had formed a normal bi-polar spindle. However, in some cells more than two poles were seen, and this was more frequent in the *Tgif1* null MEFs, but increased in both genotypes with increasing passage number (for example, see Figure 1C). To determine whether *Tgif1* null MEFs were becoming senescent, we stained for senescence associated β-galactosidase (SAβG) activity at passages 5 and 6. The *Tgif1* null MEFs had significantly more SAβG positive cells in the culture than the wild type at both passages, suggesting increased senescence in cells lacking Tgif1 (Figure 1D and E). We also tested whether the reduced ability of *Tgif1* null cultures to proliferate was due to increased apoptosis by staining with annexin V. As shown in Figure 1F, there was a small but significant increase in the number of apoptotic cells in the *Tgif1* null cultures at P5, but this represented a relatively minor proportion of the culture. When we compared expression of a number of cell cycle inhibitors by western blotting, we observed an increase in the levels of both the p27 and p19 proteins in *Tgif1* null MEFs, consistent with decreased proliferation and increased senescence (Figure 1G). Thus, it appears that premature senescence represents a major contribution to the reduced proliferation rate of MEFs lacking Tgif1.

### Increased ploidy in MEFs lacking Tgif1

**Figure 2:**
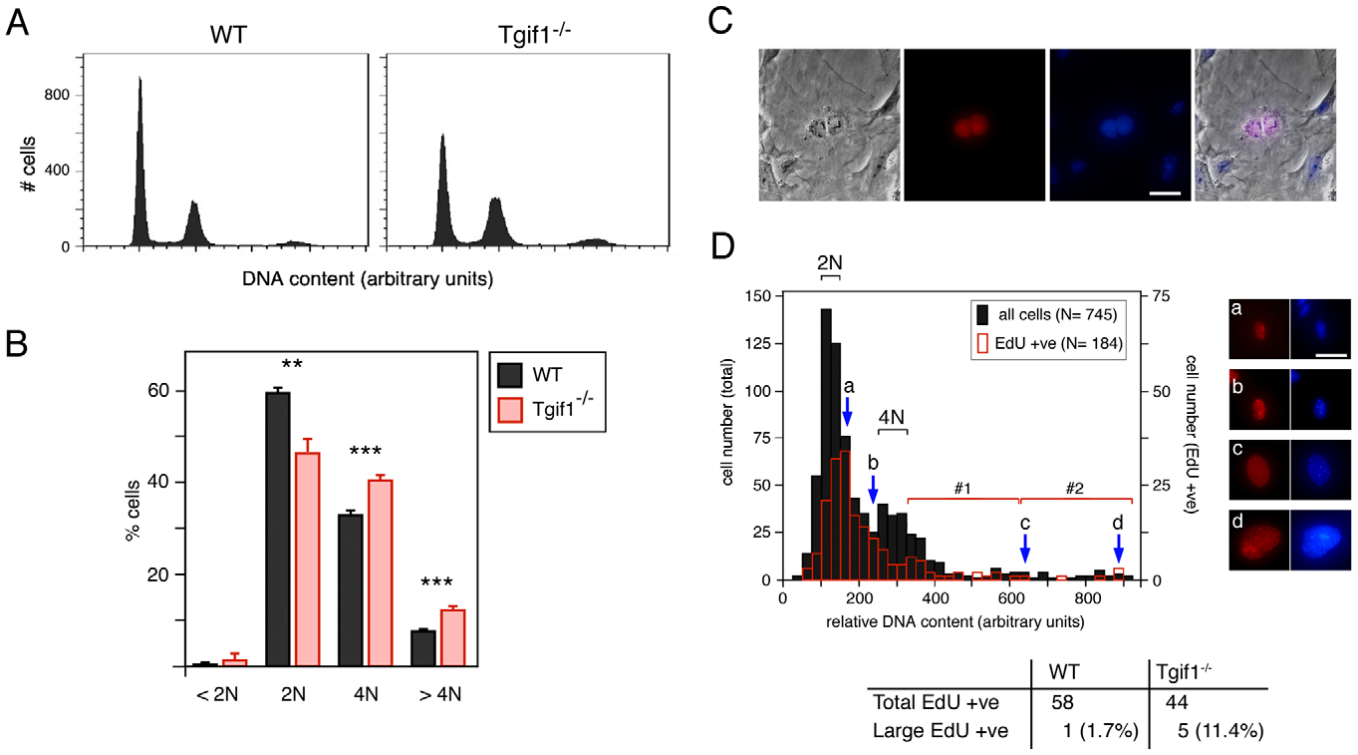
Cell cycle analysis of *Tgif1* null MEFs. A) Cell cycle profiles of wild type and *Tgif1* null MEFs at passage 4 were generated by propidium iodide staining and FACS analysis. DNA content is plotted against cell number. B) Cell cycle distribution was determined as in panel A, and the percentage of cells with 2N or 4N DNA content, as well as those with greater than 4N and less than 2N is shown, as the average + s.d of triplicate cultures. P-values determined by the Student's T test are indicated: **<0.01, ***<0.001. C and D) Wild type and *Tgif1* null MEFs were incubated with EdU (for 1 hour) and stained with Hoechst, and images were captured at 10× magnification in Openlab. A representative image of an EdU positive binucleate cell is shown (C): From left to right: Phase contrast image, EdU staining (red), Hoechst stain for DNA (blue), and an overlay of all three images. D) Relative DNA content, visualized by Hoechst stain, was determined in Openlab and is plotted against cell number (black bars, 745 cells in total). The open red bars indicate the number of EdU positive cells (of 184 total) with the indicated DNA content as determined by Hoechst staining. The approximate positions of 2N and 4N DNA content peaks are indicated. Arrows (a, b, c, d) indicate the position on the profile of the representative cells shown to the right. The region of the profile containing cells with greater than 4N DNA content was divided in half (brackets #1 and 2). EdU positive cells with DNA contents that fall into bracket 2 in the cell cycle profile were quantified as a percentage of the total EdU positive population for each genotype. Scale bars = 100 µM.

To determine whether there was altered ploidy in the *Tgif1* null cultures, we stained cells with propidium iodide and analyzed them by flow cytometry (Figure 2A). We observed a significantly higher proportion of both 4N and 8N cells in the *Tgif1* null cultures, with a reduction in the 2N peak (Figure 2A, B). Additionally there was a small increase in sub-2N cells in the *Tgif1* null cultures, indicative of apoptosis (Figure 2B). The increase in 4N cells could represent cells which have arrested during G2/M, but could also include tetraploid G1 cells. The 8N population is likely to come from 4N cells that have failed to divide and have then re-replicated their DNA in the subsequent cell cycle. To examine the mechanism by which 8N cells arise, P4 MEFs were labeled with EdU for 1 hour, stained with Hoechst and then visualized by fluorescence microscopy. We found relatively few EdU positive bi-nucleate cells in either the wild type or *Tgif1* null cultures, suggesting that failure of cytokinesis after successful nuclear division was not a major route by which they become tetraploid (see Figure 2C for an example of a rare EdU positive bi-nucleate cell). To examine the alternate possibility, that *Tgif1* null cells failed both nuclear and cellular division, we analyzed images of more than 700 cells for nuclear area and Hoechst fluorescence intensity, as a measure of DNA content. This data was used to generate a cell cycle profile that had clearly distinguishable peaks representing 2N and 4N DNA content (Figure 2D). The majority of cells which were replicating their DNA (EdU positives) fell between the 2N and 4N peaks, as expected (Figure 2D). However, we also identified a number of EdU positive cells which appeared to have much higher DNA content (for example, cells c and d; Figure 2D). To quantify this we separated the area of the distribution to the right of the 4N peak into two halves and counted only the largest cells (bracket #2, Figure 2D) as being outside the normal range. At passage 4 more than 10% of EdU positive *Tgif1* null cells fell into this group, suggesting that in the absence of Tgif1, cells that become tetraploid do so primarily due to a failure of nuclear division.

### Increased DNA damage in *Tgif1* null MEFs

**Figure 3:**
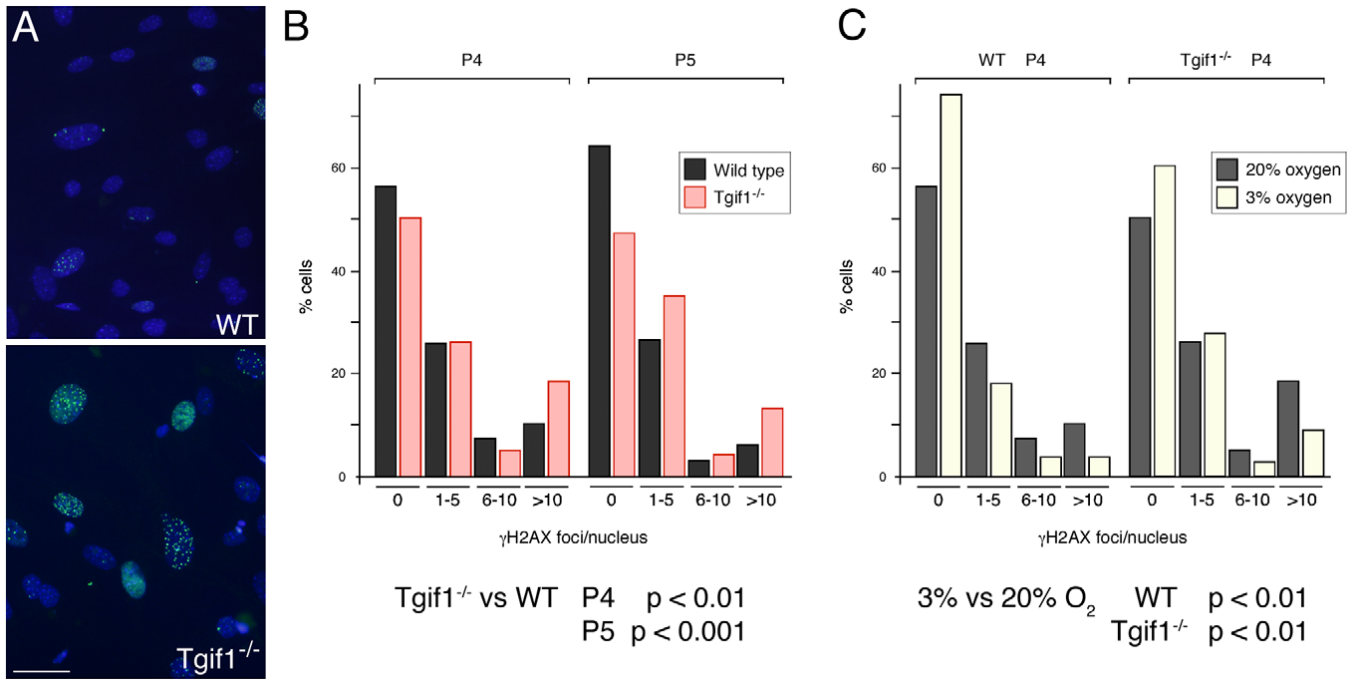
*Tgif1* null MEFs have increased DNA damage foci. A) Representative images of passage 5 cells stained with an antibody against γH2AX (green) and Hoechst (blue) are shown. Images were captured at 20×. Scale bar = 50 µM. B) The distribution of the number of γH2AX damage foci per nucleus is shown for passage 4 and 5 wild type and *Tgif1* null cells. Significance values were determined by Chi squared test, comparing the distribution in *Tgif1* null to that expected based on the wild type. C) The distributions of the number of γH2AX damage foci per nucleus in passage 4 cells were compared to those from cells that had been grown in 3% oxygen from passage 2 to 4. Data is presented and analyzed as in B. Note that the P4 MEFs analyzed for damage foci in 20% oxygen in panels B and C are the same. p-values for the comparisons of wild type to *Tgif1* null and 3% to 20% oxygen are shown below.

One of the stresses imposed on MEFs in culture is growth under hyperoxic conditions – the 20% oxygen atmosphere of cultured cells compared to around 3–5% oxygen which cells in the animal experience. Growth in high oxygen can result in increased DNA damage, which in turn can contribute to the onset of cellular senescence [27]. To test whether there was increased DNA damage in *Tgif1* null MEFs we stained cells with an antibody that recognizes phosphorylated H2A.X (γH2AX), which is found at repair foci. In *Tgif1* null MEFs, particularly at later passages, we observed an increase in the proportion of cells with large numbers of damage foci (for example, see Figure 3A). To quantify possible differences in numbers of damage foci, we grouped cells by the number of γH2AX foci per nucleus (no foci, 1–5, 6–10, or >10 foci per nucleus), and compared the distributions of more than 200 wild type and mutant cells. At passages 4 and 5, there was a significant shift towards cells with higher numbers of damage foci in the mutant cultures, with an increase in the proportion of *Tgif1* null cells with more than 10 γH2AX foci per nucleus and a decrease in the number of cells with no foci (Figure 3B). To test whether the increase in DNA damage foci was due to hyperoxia, we cultured cells under regular tissue culture conditions (20% oxygen) or in normoxic conditions (3% oxygen) and compared the numbers of γH2AX foci per nucleus. As shown in Figure 3C, there was a significant decrease in the number of foci per nucleus for both wild type and *Tgif1* null cells when cultured in 3% oxygen.

**Figure 4:**
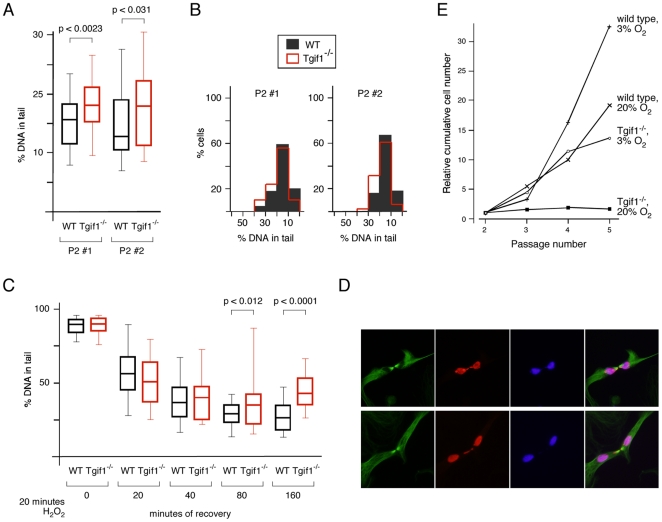
Increased DNA damage in MEFs lacking Tgif1. Passage 2 wild type and *Tgif1* null cells were analyzed by comet assay, under denaturing conditions. The percentage of total DNA in the tail was quantified for at least 50 cells per condition. A) The percentage of DNA in the tail is plotted for each of two independent batches of wild type and *Tgif1* null MEFs. Data is plotted as median, 25^th^ and 75^th^ percentiles (box) and 5^th^ and 95^th^ percentiles (whiskers). p-values determined by the Student's T test are shown. B) The data shown in A, binned into 5% blocks, are plotted to show the distribution. C) Cells were treated with 100 µM H_2_O_2_ for 20 minutes and analyzed by comet assay at time-points thereafter over a 160 minute time-course. Data are presented as in A, with p-values for comparisons between wild type and Tgif1 null shown where significant. D) representative images of mitotic cells with DNA bridges are shown for *Tgif1* null cells. Cells analyzed for α-tubulin (green), pHH3 (red), and Hoechst (blue) are shown, together with a merged image of all three colors. Images were captured at 40×. Scale bar = 25 µM. E) Wild type and *Tgif1* null MEFs were grown on a 3T3 protocol in a standard incubator (5% CO_2_ in air [20% O_2_]), or in a chamber with 5% CO_2_ and 3% O_2_. Growth is plotted as relative cumulative cell number, with the starting 300,000 cells at P2 set equal to 1.

To directly assess the level of DNA damage we performed comet assays under denaturing conditions, to detect both single and double strand breaks. There was significantly more DNA damage in the *Tgif1* null cells compared to the wild types at passage 2 (Figure 4A). Comparison of the distribution of the amount of damaged DNA per nucleus revealed a shift in the overall distribution of damage between wild type and mutants, rather than the presence of a sub-population with much higher levels of damage (Figure 4B). We next analyzed recovery from H_2_O_2_ induced DNA damage using the comet assay. Passage 2 cells were exposed to H_2_O_2_ for 20 minutes and the amount of DNA damage scored immediately, or at time-points thereafter. After only 20 minutes of recovery there was a clear decrease in the amount of damaged DNA detected by this assay, and the wild type and mutant cells were not significantly different at this point (Figure 4C). However, at later time-points we observed significantly more residual damage in the *Tgif1* null cells compared to the wild type (Figure 4C). Analysis of the distribution of cells with different amounts of damage suggests that in the *Tgif1* null cells, there is a general shift in the distribution as seen in undamaged cells, rather than the presence of a sub-population that fails to repair (data not shown). Interestingly, while analyzing cells for mitotic spindles, we also noticed that in a number of cells in which the majority of the DNA had separated to two poles, there was a DNA bridge linking the separated chromosomes (for example, see Figure 4D). These DNA bridges were seen more frequently in the *Tgif1* null MEFs and more frequently at later passages. DNA bridges have been linked to entry into mitosis without having fully repaired DNA damage, and thus may be consistent with unrepaired DNA damage resulting in changes in ploidy [35], [36]. Together, this data suggests that *Tgif1* null MEFs are less able to deal with DNA damage induced by oxidative stress. To test whether the increased DNA damage observed in *Tgif1* null MEFs contributed to the proliferation defect, we cultured cells on a 3T3 protocol under regular tissue culture conditions (20% oxygen) or in normoxic conditions (3% oxygen). Culturing *Tgif1* null MEFs in 3% oxygen resulted in an increased proliferation rate, that was close to that observed in the wild type cells grown under standard tissue culture conditions (Figure 4E). Additionally, there was an increase in proliferation of the wild type MEFs under these conditions, suggesting that both wild type and *Tgif1* null MEF proliferation is reduced by hyperoxia. Together this data suggests that *Tgif1* null MEFs have an increased level of DNA damage, that is due in part to the hyperoxic stress of culture, and that hyperoxic stress contributes to the reduced proliferation in the absence of Tgif1.

### Transcriptional changes in *Tgif1* null MEFs

**Figure 5:**
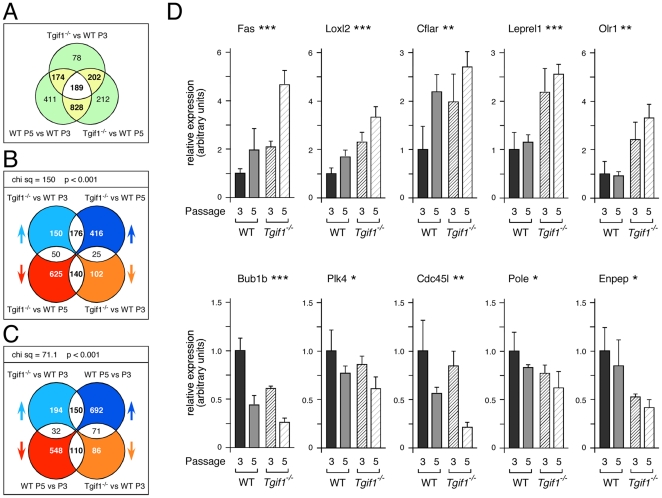
Global analysis of transcriptional changes. A) RNA from three sets of triplicate cultures (passage 3 wild type [P3], passage 5 wild type [P5], and passage 3 *Tgif1* null MEFs [null]) and was analyzed on Affymetrix expression arrays. A Venn diagram is shown with the numbers of probe-sets that changed significantly in each of three pair-wise comparisons (null – P3, null – P5, and P5 – P3). B) An analysis of the overlap between the null – P3 and null – P5 comparisons is shown as a four-way Venn diagram, allowing overlaps between a maximum of two data-sets. The arrows indicate the direction of the change in signal: For example, pale blue arrow indicates increased signal in the null – P3 comparison. Of the 376 probe-sets that increased in the null – P3 comparison, 176 increased and 50 decreased in the null – P5 comparison, whereas 150 did not change significantly in the null – P5. The distribution of changes in the probe-sets present in the overlaps (176, 25, 50, 140) was analyzed using a 2×2 contingency table and a chi squared test. The chi squared value and p-value are shown above. C) An analysis of overlap of the data from the null – P3 and P5 – P3 comparisons is shown, as in panel B. D) Expression of ten genes from the overlaps shown in B and C was analyzed by qRT-PCR in RNAs from triplicate cultures of wild type and *Tgif1* null MEFs at both passage 3 and 5. Expression is presented as the average (+ s.d.) in arbitrary units with the P2 wild type set equal to 1 for each gene. Significance levels as determined by ANOVA are indicated above (* p<0.05, ** p<0.01, *** p<0.001).

For the phenotypes described so far, the wild type MEFs display similar defects to the *Tgif1* null, either at a lower rate or a later passage, suggesting that *Tgif1* null MEFs undergo the same crisis as wild type MEFs, but at an earlier passage number. To further test this possibility we performed expression array analysis of wild type MEFs at passages 3 and 5 and *Tgif1* null MEFs at passage 3. We reasoned that if *Tgif1* null MEFs undergo a similar crisis to the wild type cells, but at an accelerated rate, then there should be an overlap in the transcriptional profiles of P5 wild type and P3 *Tgif1* null MEFs. RNA was isolated from three independent cultures for each of the three cell types (P3 and P5 wild type and P3 *Tgif1* null) and analyzed on Affymetrix arrays. Data was filtered using a 0.0001 p-value cut-off and a log-fold change of +/−0.5 in any one of three pair-wise comparisons: P5 – P3, *Tgif1* null – P3, and *Tgif1* null – P5. 2094 probe-sets were selected by this cut-off (Table S1). Using a more stringent log-fold change cut-off of +/−1.0, and removing duplicate and unannotated probe-sets, the number of genes that increased by at least 2-fold in the *Tgif1* null was 78, and only 37 decreased by more than 2-fold (Table S2). Among the genes that increased by more than two-fold, there was an enrichment for genes involved in muscle development, including four troponin genes and four myosin genes. We next identified which probe-sets changed in two of the three pair-wise comparisons. The majority (363/643; 56%) of probe-sets that were different between *Tgif1* null and P3 wild-type MEFs were also different between P5 and P3 wild types (Figure 5A). As expected, there was also a significant overlap between probes that changed in the *Tgif1* null compared to P3 wild types and to P5 wild types. Comparing the *Tgif1* null to both P3 wild type and P5 wild type data-sets, showed that the majority of changes were in the same direction in both comparisons. Thus 176 probe-sets (45%) out of the total overlap of 391 increased in the *Tgif1* null relative to both P3 and P5 wild type cells, and 140 (36%) decreased in both comparisons (Figure 5B). To test whether the distribution of changes among the overlap was significantly different from that expected by chance, we used a 2×2 contingency table and chi squared test. As shown in Figure 5B, the enrichment for probe-sets that increased or decreased in both comparisons was highly significant.

To identify the types of genes represented within these changes, we used the DAVID functional annotation tool (http://david.abcc.ncifcrf.gov) [37], [38] to assign GO terms to the probe-sets that changed in the comparison of *Tgif1* null to wild type P3 MEFs. Clusters for probe-sets with increased signal in the *Tgif1* null represented genes involved in muscle cell development and differentiation, and cell adhesion and apoptosis (Table S3). Consistent with the decreased proliferation in the *Tgif1* null MEFs, probe-sets with decreased signal in the *Tgif1* null were enriched for genes involved in cell cycle progression, mitosis and DNA replication (Table S3). To further categorize the potential Tgif1-specific changes, we performed clustering analysis on the probe-sets that either increased or decreased in the comparison of *Tgif1* null to both P3 and P5 wild type cells. Cell adhesion and cytoskeletal functions remained prominent in the clusters that increased, whereas those that decreased showed much less significant changes, suggesting that many probe-sets with decreased signal in the *Tgif1* null cells overlap with changes in the later passage wild type MEFs (Table S4).

We next examined probe-sets that changed significantly with both increasing passage and with loss of Tgif1. Of the 363 probe-sets that changed in both P5 wild type and *Tgif1* null MEFs compared to the P3 control, the majority had an increased signal in both, or a decreased signal in both (Figure 5C; 150/363 up in both, and 110/363 down in both). There was an enrichment for genes involved in DNA replication, cell cycle progression and mitosis in the 110 probe-sets that decreased in both P5 MEFs and P3 MEFs lacking Tgif1, whereas, genes with links to apoptosis were enriched among probe-sets with increased signal in both comparisons (Table S5). This analysis suggests that the major overlap between transcriptional changes in P5 MEFs and *Tgif1* null MEFs represents genes involved in cell cycle progression and cell death, consistent with the proliferation defects in *Tgif1* null MEFs. We next selected a panel of ten genes represented by probe-sets that changed either in a Tgif1-specific manner or dependent on both passage and loss of Tgif1 and analyzed expression by qRT-PCR. For this analysis we also included RNAs generated from P5 *Tgif1* null MEFs. While many of the probe-sets representing cell cycle related genes that were reduced in both the P5 and *Tgif1* null MEFs had relatively modest changes (−0.5 to −0.7 log), we were able to verify statistically significant changes in expression by qRT-PCR (Figure 5D). For example, *Loxl2* expression increased with passage and with loss of Tgif1 and *Bub1b* decreased in both conditions. In contrast, *Leprel1* and *Olr1* appeared to be Tgif1-specific; their expression increased in *Tgif1* null MEFs but was not affected by passage (Figure 5D). Taken together, this analysis suggests that there is considerable overlap between the changes in MEFs lacking Tgif1 and in MEFs at later passage, consistent with the notion that *Tgif1* null MEFs are undergoing a similar crisis to later passage wild type cells.

### Proliferation defects and gene expression changes with transient Tgif1 reduction

**Figure 6:**
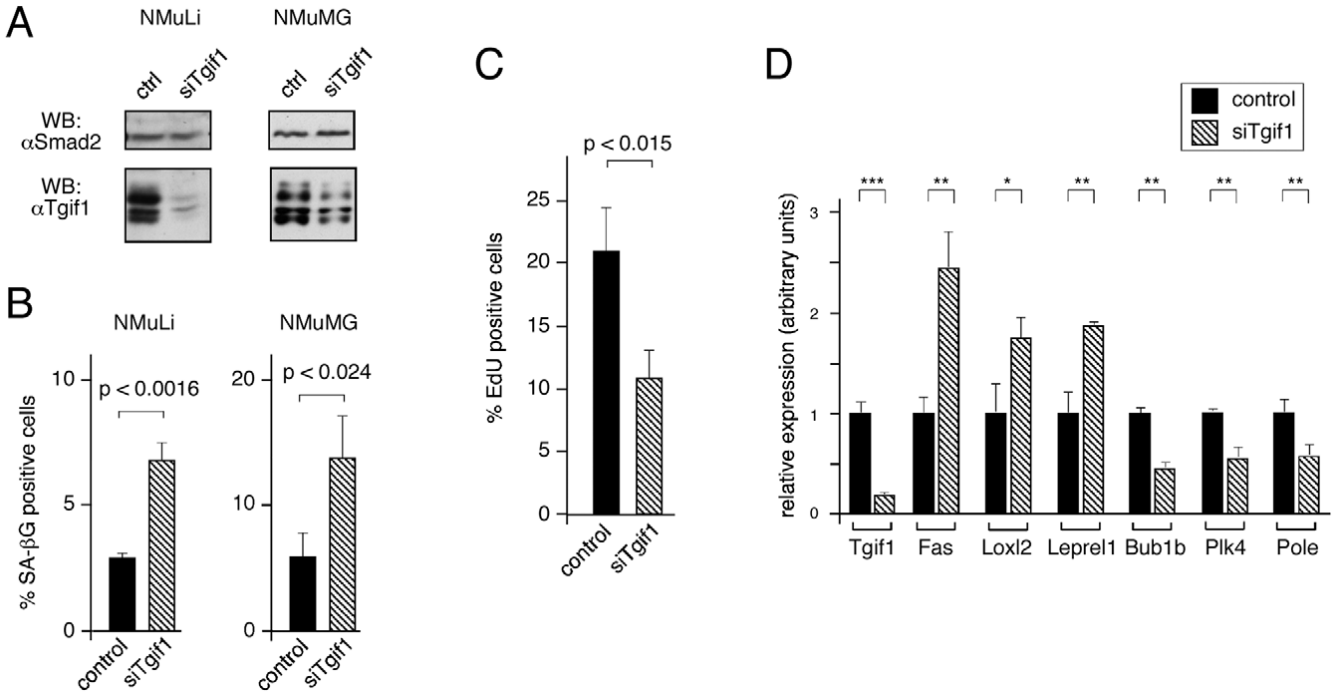
Proliferation defects with transient knock-down of *Tgif1* in NMuLi cells. A) NMuLi and NMuMG cells were transfected with siRNAs targeting *Tgif1*, or with a control pool, and Tgif1 protein levels were analyzed 48 hours later, by western blot. Smad2 levels are shown as a loading control. B) Control and *Tgif1* knock-down NMuLi and NMuMG cells were analyzed for senescence associated β-gal staining 72 hours after knock-down. The percentage of SA β-gal positive cells is presented as mean + s.d. of triplicate transfections, together with p values. C) Control and *Tgif1* knock-down NMuLi cells were analyzed for EdU incorporation, as a measure of proliferation. Cells were incubated with EdU for 1 hour, 48 hours after transfection. Data is presented as mean + s.d. of triplicate transfections, together with the p value. D) Expression of the ten genes analyzed in Figure 5D was tested in control and *Tgif1* knock-down NMuLi cells by qRT-PCR from triplicate cultures. Data is shown for *Tgif1* and the six genes for which differences in expression were significant. All p values were determined by the Student's T test (* p<0.05, ** p<0.01, *** p<0.001, for panel D).

Given the similarity between the effects of loss of Tgif1 and increased passage in MEFs, we wondered whether any similar effects of reducing Tgif1 levels might be seen in other cell types. To determine whether the effects of loss of Tgif1 function on proliferation and gene expression were limited to primary MEFs we tested the effects of transient knock-down of *Tgif1* in the mouse liver cell line, NMuLi, and in the normal murine mammary gland cell line, NMuMG. We have previously shown that knocking down *Tgif1* in NMuLi cells affects expression of a sub-set of nuclear receptor regulated genes, suggesting that they may be a good model in which to test effects of Tgif1 [26]. We transiently knocked-down *Tgif1* and isolated protein 48 hours later, or analyzed parallel cultures for SAβG staining at 72 hours (Figure 6A and B). In both NMuLi and NMuMG we observed a small but significant increase in SAβG staining in the *Tgif1* knock-down at 72 hours post-transfection (Figure 6B). Since the knock-down in NMuLi cells appeared to be more efficient, we also analyzed proliferation and gene expression in these cells. When control and *Tgif1* knock-down NMuLi cells were incubated with EdU to monitor DNA sysnthesis, we observed an almost two-fold decrease in the number of cells incorporating EdU in the *Tgif1* knock-down cultures (Figure 6C). Thus, it appears that reducing Tgif1 levels results in both decreased proliferation and increased senescence in immortalized cells as well as in primary MEFs. To further probe the similarity between the effects of *Tgif1* knock-out and knock-down we analyzed expression of the panel of ten genes tested based on the array data (see Figure 5D). As shown in Figure 6D, six of the ten genes tested showed significant changes in expression in NMuLi cells with reduced Tgif1 expression. Thus it appears that there is good concordance between the effects of complete loss of Tgif1 function in primary MEFs and a transient reduction in Tgif1 expression levels in an immortalized cell line.

### A role for TGFβ signaling in the growth defect

**Figure 7:**
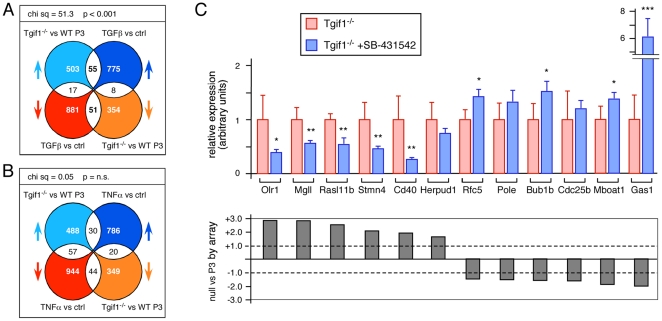
Overlap of TGFβ-mediated transcriptional changes with those in *Tgif1* null MEFs. A) Data from the comparison of *Tgif1* null to wild type P3 MEFs was compared to that from MEFs treated with TGFβ for 10 hours (from GSE15871). Total numbers of probe-sets with significant changes, and the overlaps are shown as in Figure 5. Chi squared analysis was performed as in Figure 5 and is shown above. B) Data from the comparison of *Tgif1* null to wild type P3 MEFs was compared to MEFs treated with TNFα for 4 hours (from GSE3700). Data was analyzed and is presented as in A. C) Twelve genes represented in the overlap shown in panel B were analyzed by qRT-PCR. Six genes each were selected from those that went up in both and those that went down in both. Expression was analyzed in triplicate cultures of passage 3 *Tgif1* null MEFs treated with a TGFβ receptor kinase inhibitor (1 µM SB-431542), or left untreated. Data is shown as mean (+ s.d.) with the value in the untreated cells set to 1 in each case. * p<0.05, ** p<0.01, *** p<0.001, as determined by the Student's T test. Shown below is the fold change (on a linear scale) in the comparison of the *Tgif1* null to wild type P3 array data for each gene.

To identify pathways that changed in the absence of Tgif1 we compared our array data from the comparison of wild type and *Tgif1* null P3 MEFs (with a significance cut-off of 0.001) to publicly available data sets. One data set of interest (GSE15871) included wild type MEFs treated with TGFβ for 1 or 10 hours, and for comparison we also analyzed data from GSE3700 in which MEFs had been treated with TNFα for 4 hours. Although only 14.6% of genes that changed in our *Tgif1* null to wild type comparison also changed in the control versus 10 hour TGFβ treatment from GSE15871, there was a significant enrichment for genes that showed either increased (55/131) or decreased (51/131) expression in the absence of *Tgif1* and in MEFs treated with TGFβ (Figure 7A). In contrast, there was no such enrichment when comparing the data from TNFα-treated MEFs with *Tgif1* null MEFs (Figure 7B). As with the overlap between *Tgif1* null and P5 wild type MEFs, probe-sets representing genes involved in cell cycle progression and DNA replication were enriched among those that decreased in both TGFβ treated and *Tgif1* null MEFs (Table S6). This analysis raised the possibility that the altered expression of a subset of genes in the *Tgif1* null MEFs was due to increased activity of the TGFβ pathway. To test this we analyzed expression of a panel of genes in *Tgif1* null MEFs treated with a TGFβ receptor kinase inhibitor (SB-431542; [39]). For five of the six genes for which there was increased expression in the *Tgif1* null and with TGFβ treatment from GSE15871, there was a significant reduction in expression when we inhibited the type I TGFβ receptor (Figure 7C). Conversely, TGFβ receptor inhibition significantly increased expression of four of the six genes for which the signal was reduced in both arrays (Figure 7C).

**Figure 8:**
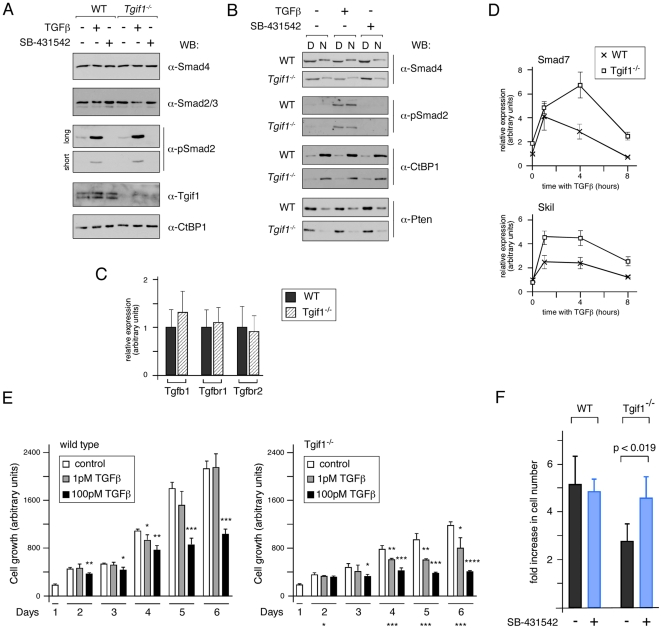
*Tgif1* null MEFs are sensitive to TGFβ mediated growth inhibition. A) Wild type and *Tgif1* null MEFs were incubated with TGFβ or SB-431542 for 1 hour as indicated, and analyzed by western blot. CtBP1 and Smad4 act as loading controls for the Smad2/3 blot. Two exposures of the phospho-Smad2 blot are shown, to allow comparison of the basal and induced phosphorylation levels. B) Western blot analysis for phospho-Smad2 of untreated and TGFβ or SB-431542 treated cells that had been separated into soluble cytoplasmic [D] and nuclear [N] fractions by sequential permeabilization with digitonin and NP40 is shown. CtBP1 (primarily nuclear) and Smad4 and Pten (both predominantly cytoplasmic) act as a fractionation controls. C) Expression of the genes encoding TGFβ1 and the TGFβ type I and type II receptors was analyzed by qRT-PCR. D) Wild type and *Tgif1* null MEFs were incubated with TGFβ for the indicated times and expression of the TGFβ-responsive *Smad7* and *Skil* genes was analyzed by qRT-PCR. E) Wild type and *Tgif1* null cells were analyzed for growth by Alamar Blue assay, daily over a 6 day period. Cells were incubated with 1 pM or 100 pM TGFβ, or without TGFβ, as indicated. Relative cell growth is shown as the average plus s.d. of triplicate wells. The significance level was determined by Student's T test, and is shown above each column for comparison to the appropriate control cultures on each day. A significant difference in growth between untreated wild type and *Tgif1* null cultures is indicated below the right hand graph. *<0.05, **<0.01, ***<0.001, ****<0.0001. F) *Tgif1* null or wild type MEFs (quadruplicate cultures) were grown on a 3T3 protocol and from P2 to P3 were treated with 0.2 µM SB-431542 twice. The relative increase in cell number (average + s.d.) is plotted for each. The p-values (determined by Student's T test) are shown for the *Tgif1* null cells treated with inhibitor, compared to the no treatment control. Differences between the wild type cultures were not significant.

In addition to acting as a Smad transcriptional corepressor [22], [40], Tgif1 has been suggested to inhibit TGFβ signaling by other mechanisms, including targeting Smad2 for ubiquitin-mediated degradation and sequestering cPML to the nucleus [41], [42]. To test whether loss of Tgif1 resulted in changes in activated Smad2 levels in primary MEFs, we analyzed Smad2 phosphorylation in response to TGFβ signaling. The overall levels of Smad2, Smad3 and Smad4 proteins were not different between wild type and *Tgif1* null MEFs, whether treated with TGFβ or SB-431542 (Figure 8A). Importantly, when we analyzed the amount of receptor-phosphorylated Smad2 seen in control and *Tgif1* null MEFs, we did not observe any increase total phospho-Smad2 in the *Tgif1* null either at basal levels or in the presence of added TGFβ (Figure 8A). We next fractionated cells into digitonin and NP40 soluble fractions representing soluble cytoplasmic and nuclear fractions. As shown in Figure 8B, phospho-Smad2 was seen in both fractions in the presence of TGFβ, and the distribution of phospho-Smad2 between nuclear and cytoplasmic fractions was not different between wild type and *Tgif1* null cells. We also tested whether loss of Tgif1 might up-regulate expression of the genes encoding TGFβ1 or its receptors, but found no significant changes in expression, consistent with the lack of increase in phospho-Smad2 levels (Figure 8C). Despite the lack of effect of Tgif1 deletion on Smad2 phosphorylation and localization, we did observe an increase in the TGFβ transcriptional response in *Tgif1* null MEFs (Figure 8D). This is clearly consistent with the role of Tgif1 as a Smad transcriptional corepressor, and raises the possibility that excess Smad2/3 transcriptional activity may contribute to the proliferation defect in *Tgif1* null MEFs.

To test whether *Tgif1* null MEFs were more sensitive to TGFβ-mediated growth inhibition we used an Alamar blue fluorimetric assay [43], [44]. Passage 2 cells were incubated with 1 pM or 100 pM TGFβ, or without ligand for up to 6 days and relative proliferation scored each day. As shown in Figure 8E, wild type cells were effectively growth inhibited by the higher dose of TGFβ, whereas 1 pM TGFβ had no significant effect. In contrast, significant growth inhibition of *Tgif1* null MEFs by both doses of TGFβ was evident from day 4 onwards (Figure 8E). 1 pM TGFβ resulted in up to 35% growth inhibition of *Tgif1* null cells, suggesting that they are more sensitive than wild type MEFs to the growth inhibitory effects of TGFβ. We next tested the possibility that increased activity of the TGFβ/Smad pathway contributes to the growth defect in the absence of added TGFβ. *Tgif1* null or wild type MEFs were cultured under a 3T3 protocol, and from P2 to P3 were incubated with or without a TGFβ receptor kinase inhibitor (SB-431542), and the increase in cell number was determined. We observed a significant increase in proliferation in the *Tgif1* null MEFs treated with the receptor kinase inhibitor, whereas no significant change in growth of the wild type cultures was observed (Figure 8F). This data suggests that an increase in the basal transcriptional output of the TGFβ/Smad pathway may contribute to the altered gene expression profile and proliferation defects in cells lacking Tgif1.

### A role for TGFβ signaling in senescence

**Figure 9:**
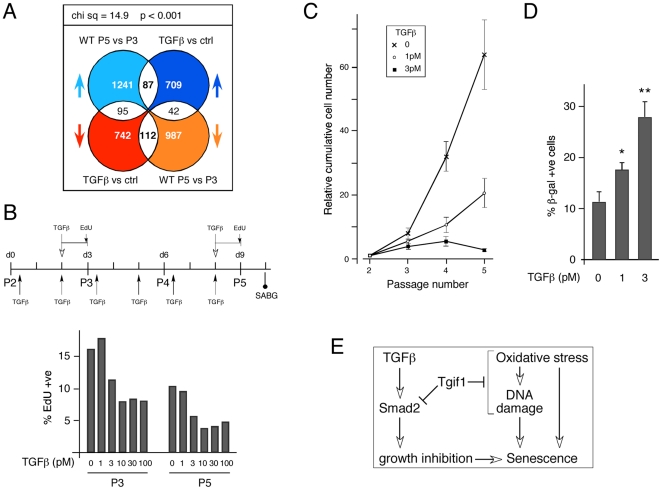
TGFβ induces growth inhibition and senescence. A) The TGFβ data-set from GSE15871 was compared to probe-set changes between P3 and P5 wild type MEFs. Data was analyzed and is presented as in Figures 5 and 7. B) The culture conditions over passages 2 to 5 are shown schematically: Arrows below indicate times at which TGFβ (1 pM or 3 pM) was added for the 3T3 assay. The time of addition of TGFβ and EdU is shown above, and the time at which the SAβG assay was performed is shown below. Relative cell proliferation was measured at passage 3 and 5 in cells grown under standard conditions, followed by a single 24 hour treatment with TGFβ at the indicated concentration. The percentage of EdU positive cells is shown. C) Cell proliferation was determined in a 3T3 assay and is shown as cumulative increase in cell number. Cells were cultured under standard conditions or with the addition of 1 or 3 pM TGFβ at the times indicated in panel B. D) The percentage (average + s.d. of triplicate cultures) of wild type cells with positive SAβG staining is shown at passage 5, after continued treatment with 1 pM or 3 pM TGFβ, or under standard conditions. Significant differences between control and plus TGFβ are shown: * p<0.05, ** p<0.01, as determined by the Student's T test. E) A tentative model describing the involvement of Tgif1 and TGFβ in the pathways leading to cellular senescence.

Comparison of the transcriptional changes between TGFβ treated MEFs (GSE15871) and our P3 to P5 wild type data set revealed an enrichment for genes that increased or decreased in both TGFβ treated and later passage MEFs, and this distribution was significantly different from random (Figure 9A). Pathway analysis revealed a significant enrichment for genes involved in cell cycle and DNA replication among probe-sets that decreased in both comparisons (Table S7). Given the overlap in transcriptional profiles between increasing passage number and TGFβ treatment, we next considered the possibility that persistent low level TGFβ stimulation might both decrease growth and promote senescence in wild type cells. We therefore cultured wild type MEFs on a 3T3 protocol and added low doses of TGFβ (1 pM or 3 pM) twice per passage (see Figure 9B). Additionally, we tested the effects of acute treatment with a range of concentrations of TGFβ at P3 and P5, on wild type MEFs grown without continual stimulation. As shown in Figure 9B, there was no effect of 24 hour treatment with 1 pM TGFβ at either passage, consistent with the lack of effect of this dose of TGFβ on wild type cells seen in the Alamar Blue assay (Figure 8D). 3 pM or higher doses resulted in some growth inhibition, which was maximal by 10 pM (Figure 9B). Analysis of the cumulative cell numbers from a 3T3 assay revealed a significant decrease in proliferation of wild type cells exposed to repeated treatment with either 1 pM or 3 pM TGFβ, despite the lack of an apparent effect of 1 pM TGFβ in shorter term assays (Figure 9C). Thus, persistent exposure to low level stimulation with TGFβ appears to be able to mimic the effect of loss of Tgif1 in a 3T3 assay. Our previous data show that at passages 5 and 6 there is an increase in the number of SAβG positive cells in *Tgif1* null compared to wild type cultures (see Figure 1). When we analyzed wild type cells at passage 5 following repeated addition of TGFβ, we observed a significant increase in the proportion of SAβG positive cells in the cultures that had been exposed to persistent low dose treatment with TGFβ (Figure 9D). This suggests that prolonged low level stimulation by TGFβ can both slow the proliferation of MEFs and induce senescence.

## Discussion

We show that primary MEFs lacking the transcriptional corepressor, Tgif1, have a compromised ability to proliferate. This appears to be due to a combination of increased activity of the TGFβ signaling pathway and increased sensitivity to oxidative stress, which together contribute to an increase in cellular senescence in MEFs lacking Tgif1 (Figure 9E). Additionally, we show that a short-term reduction in Tgif1 levels in immortalized cells causes decreased proliferation and increased senescence, suggesting that the effects of Tgif1 are not limited to primary MEFs.

The best characterized role of Tgif1 is as a repressor of TGFβ signaling [22], [40], although there is evidence for other functions of Tgif1 in the TGFβ pathway [41], [42]. Consistent with the increased TGFβ transcriptional output in *Tgif1* null MEFs, some of the defects observed in early embryos lacking both Tgif1 and Tgif2 have been shown to be partially rescued by reducing the dose of Nodal, clearly suggesting an *in vivo* role for Tgifs in the response to TGFβ family ligands [20]. Our analysis of components of the TGFβ-Smad pathway in *Tgif1* null MEFs suggests that regulating the levels of active Smad2 is not the major TGFβ pathway function of Tgif1 in primary MEFs, since we do not observe effects on Smad phosphorylation or localization. We cannot definitively rule out a contribution of such effects to the phenotypes observed in *Tgif1* null MEFs. A more detailed analysis of how Tgif1 controls TGFβ pathway output would clearly be of interest, and it remains possible that different mechanisms might function in different cells types, or at different points in the course of the TGFβ response. Additionally, it is possible that loss of Tgif1 might alter expression of components of the TGFβ pathway, thereby indirectly affecting pathway output. However, our analysis of *Tgfb1* and TGFβ receptor gene expression, as well as the lack of change in phospho-Smad2 levels, suggests that this is not a major contributor to the *Tgif1* null phenotype.

In many cell types, including epithelial cells, thymocytes and primary MEFs, TGFβ signaling promotes a G1 cell cycle arrest. In the human keratinocyte cell line, HaCaT, over-expression of Tgif1 reduced the anti-proliferative effect of TGFβ [45]. Here we show that MEFs lacking Tgif1 are more sensitive to TGFβ-mediated growth inhibition. *Tgif1* null MEFs proliferate less well, and when treated with a TGFβ type I receptor kinase inhibitor, there is a significant increase in proliferation of the *Tgif1* null cells. This suggests that there is a TGFβ/Smad-dependent component to the reduced proliferation in *Tgif1* null MEFs. It should be noted here that although culturing *Tgif1* null MEFs in the presence of the receptor kinase inhibitor for three days increased proliferation, longer term incubation (over more than one passage) resulted in a dose dependent decrease in proliferation of both wild type and *Tgif1* null cells. Together with the effect of Tgif1 knock-down in NMuLi cells shown here, and the previous demonstration that Tgif1 expression of can attenuate TGFβ mediated growth inhibition, these results suggest that Tgif1 is a key regulator of the anti-proliferative effects of TGFβ signaling. However, in myeloid cells Tgif1 knock-down decreased proliferation, without an increase in G1 cells, as would be expected with a TGFβ mediated cell cycle block [46], raising the possibility of an additional TGFβ independent role for Tgif1 in regulating proliferation. This is supported by our data, which suggest that there are additional effects of loss of Tgif1, which result in increased sensitivity to oxidative stress and increased DNA damage.

The defects in *Tgif1* null MEFs include a reduced ability to deal with DNA damage and premature induction of a senescent phenotype. Despite the increase in senescence in *Tgif1* null MEFs, there is no strong evidence for a premature aging phenotype in mice lacking Tgif1, and because mice lacking both Tgif1 and Tgif2 are not viable [20], [48] it is not possible to test whether loss of both proteins causes aging phenotypes in mice. *Tgif1* null mice in a C57BL/6 strain background show some growth retardation [19], and an increased frequency of hydrocephalus and kyphosis (data not shown). Whether these are truly aging related phenotypes remains to be determined, since placental defects may contribute to the growth retardation, and the frequency of other possible aging related phenotypes is quite low. However, it should be noted that the penetrance of senescent phenotypes even at the cellular level is quite variable [47]. Our data suggest that the increase in DNA damage in *Tgif1* null cells is due at least in part to the hyperoxic stress of being placed in culture. These phenotypes are characteristic of advanced passage wild type primary MEFs, but appear to be more severe in *Tgif1* null cells. Importantly, we show that culturing cells under more physiological oxygen conditions results in partial rescue of their ability to proliferate. It should be noted, however, that wild type MEFs also proliferated better in 3% oxygen, such that there was still a difference between wild type and null cells. Both wild type and *Tgif1* null cells had high levels of DNA damage and γH2AX-containing repair foci in their nuclei, but the number of repair foci and the amount of DNA damage were significantly higher in the *Tgif1* nulls. Thus it appears that the *Tgif1* null cells have essentially the same defects as later passage wild type MEFs – high levels of DNA damage and premature senescence – but these defects occur earlier in cells lacking Tgif1. This suggests that Tgif1 may play a role in protecting MEFs from oxidative stress. Our attempts so far to identify the precise mechanism by which Tgif1 regulates oxidative stress have not been successful. Analysis of mitotic defects in cells grown in normoxic conditions suggests that reducing the hyperoxic stress of culture conditions can reduce the number of cells with DNA bridges and multiple spindles, suggesting a link between the DNA damage and senescence phenotypes (data not shown). Comparison of the recovery of wild type and *Tgif1* null MEFs from induced DNA damage suggests that it is not a failure to repair DNA damage, but rather a higher steady state level in the *Tgif1* null. Consistent with this, we see increased phosphorylation of the checkpoint kinase Chk1 in *Tgif1* null MEFs when cultured under standard conditions (data not shown), but we have not shown any effect of inhibiting kinases involved in the DNA damage response on proliferation or DNA damage levels in *Tgif1* null MEFs. Although our array analysis revealed an overlap between transcriptional changes in *Tgif1* null and later passage wild type MEFs, we have not yet identified specific Tgif1 gene targets that might mediate the premature senescence phenotype, a task that may be complicated by the possibility that both TGFβ- dependent and independent functions of Tgif1 could play a role.

As wild type MEFs senesce, an increasing proportion of the cells become tetraploid. Analysis of the cell cycle distribution of our *Tgif1* null MEFs suggests that there is an increase in cells with both 4N and 8N DNA content, and a corresponding decrease in the 2N population. In addition to the increased ploidy in the *Tgif1* null, there was also an increase in cells with sub-2N DNA content, suggesting that there is an increase in apoptosis in the absence of Tgif1. For cells to become tetraploid, with 8N DNA content, they must first fail cytokinesis, or both nuclear and cellular division. Our analysis suggests that relatively few bi-nucleate cells enter S phase either in wild type or *Tgif1* null cultures, and it appears that the primary way in which *Tgif1* null MEFs become tetraploid is by a failure of nuclear division. This may be due to a reduced ability to clear DNA damage during G2/M, followed by escape into G1. There is evidence that entry into mitosis without having correctly cleared DNA damage results in the formation of DNA/chromatin bridges linking the separating chromosomes [35], [36]. In such cases this may result in a failure of nuclear division and the entry of 4N cells into G1, followed by DNA replication in the subsequent S phase to generate 8N cells. Thus the growth defect in *Tgif1* null MEFs is likely due in part to an increased number of tetraploid cells which do not continue to proliferate.

In addition to the effects of hyperoxic stress, there appears to be a TGFβ-dependent component to the growth defect in *Tgif1* null MEFs, and we show that persistent low level TGFβ signaling in wild type MEFs can induce senescence, even when added at levels that do not cause significant growth inhibition in shorter term assays. Thus it appears that there is a distinct TGFβ dependent pathway that can also induce senescence. One possibility is that altered expression of cell cycle regulators increases the chance that cells exit the cell cycle and become senescent. Global gene expression analysis revealed a significant enrichment for genes that were either up- or down-regulated both by loss of Tgif1 and by increasing passage, consistent with the idea that *Tgif1* null MEFs senesce prematurely. Pathway analysis shows an enrichment for genes involved in cell cycle progression and DNA replication among those that are down-regulated by both loss of Tgif1 and passage. Similarly, there was an enrichment for genes that increased or decreased expression with both the addition of TGFβ and with increasing passage, or with the addition of TGFβ and with loss of Tgif1. This analysis is consistent with Tgif1 playing a role in regulating the basal activity of the TGFβ pathway, and with increased TGFβ signaling contributing to the senescent phenotype in MEFs. Our attempts to identify a sub-set of genes that changed similarly with increasing passage, TGFβ addition and loss of Tgif1 met with limited success. Only a relatively small group of probe-sets was identified by this triple overlap analysis, and verification of the changes predicted by the array analysis was not successful for all of them. This is consistent with the model that Tgif1 slows MEF senescence by two separate pathways, namely limiting TGFβ mediated gene expression and reducing the effect of hyperoxic stress. Careful scrutiny of the array data reveals potential changes in some genes that are clearly consistent with the phenotypes observed, but we have as yet been unable to identify specific gene expression changes that cause the change in sensitivity to hyperoxic stress in the *Tgif1* null cells.

In summary, we show that in primary MEFs, loss of Tgif1 results in reduced proliferation, due to increased activity of the TGFβ/Smad pathway and a decreased ability to cope with hyperoxic stress (Figure 9E). This suggests that Tgif1 is a regulator of TGFβ signaling, but also points to other functions for this corepressor.

## Materials and Methods

### Ethics Statement

All animal procedures were carried out as part of protocol 3026, and were approved by the Animal Care and Use Committee of the University of Virginia, which is fully accredited by the AAALAC.

### Cell culture, MEF isolation and siRNA knock-down

The *Tgif1* and *Tgif2* alleles have been described, and were maintained on a mixed C57BL/6J×129Sv/J background. MEFs were isolated from 13.5 day mouse embryos, and cultured in DMEM (Invitrogen) with 10% Fetal Bovine Serum (Hyclone). All procedures were approved by the Animal Care and Use Committee of the University of Virginia. For 3T3 assays, MEFs were seeded at 3×10^5^ cells per 10 cm plate, trypsinized after three days, counted with a hemacytometer, and re-seeded at the same density. For growth in low oxygen, MEFs were grown in a humidified hypoxia chamber that was flooded with a gas mixture of 92% Nitrogen, 3% Oxygen, 5% Carbon Dioxide. To induce DNA damage, cells were treated with 100 µM Hydrogen Peroxide for 20 minutes at 4°C. Cells were treated with the SB-431542 TGFβ type I receptor kinase inhibitor at a concentration of 0.2 µM. For Growth analysis, cells were treated twice, at 5 and 48 hours after plating. NMuLi cells were maintained in DMEM with 10% FBS. For knock-down, cells were plated in 6 well plates and transfected with Dharmacon SMARTpool oligonucleotides against *Tgif1* [26], using DharmaFECT reagent 1. The control pool (mouse siGENOME Non-targeting siRNA pool #3) was used for the non-targeting control.

### Antibodies and immunofluorescence

Cells were grown on glass coverslips, fixed at −20°C in Methanol, and permeabilized using 0.1% Triton X-100 (Sigma) in PBS for 15 minutes. Blocking was for 45 minutes in 10% FBS in PBS. For anti-γH2AX, cells were fixed in 3.7% formaldehyde and blocked with 2% Bovine Serum Albumin, 2% Newborn Calf Serum, and 0.02% Sodium Azide, and permeabilized in 0.25% Triton X-100 in PBS for 15 minutes. DNA was stained using Hoechst 33342 (Sigma). Antibodies were as follows: Mouse anti-γ-tubulin (Sigma T6557) (1∶400), rabbit anti-histone H3 phospho-serine 10 (Millipore 06-570) (1∶1000), mouse anti-α-tubulin (Sigma T9026) (1∶400), mouse anti-γH2AX (Millipore JBW301) (1∶500). Antibodies were diluted in 5% FBS and incubated for 1.5 hours at room temperature. Secondary antibodies were Alexafluor 594 anti-rabbit and Alexafluor 488 anti-mouse (Invitrogen). Images were captured on an Olympus BX51 microscope and images visualized in OpenLab and Photoshop CS2. Quantification of fluorescence intensity and area was performed in OpenLab.

### Senescence associated β-gal and annexin V staining

Cells were fixed in 20% formaldehyde, 2% glutaraldehyde in PBS at room temperature, and stained using a Senescence Associated β-Galactosidase staining kit (Cell Signaling #9860). Staining was visualized using a Leica MZ16 stereomicroscope, and images captured with a QImaging 5.0 RTV digital camera. For annexin staining, cells were washed in PBS, then in cold Annexin binding buffer (1 mM HEPES, 140 mM NaCl, 2.5 mM CaCl2, pH 7.4), and stained with Annexin V (594 conjugate, Molecular Probes, A13203) diluted in Annexin binding buffer for 15 minutes at room temperature in the dark. Images were captured on an Olympus BX51 microscope and images visualized in Photoshop CS2.

### Cell cycle analysis

Cells were fixed in 70% ethanol at 4°C. DNA was stained with Propidium Iodide (Sigma P4170) and analyzed by flow cytometry on a Becton Dickinson FACSCalibur and analyzed with FlowJO. For cell cycle profiles generated by fluorescence microscopy, cells were labeled with 10 µM EdU for 1 hour at 37°C. Following fixation in 4% paraformaldehyde, cells were permeabilized with Triton X-100 for 30 minutes at room temperature, and stained with an AlexaFluor 488 EdU detection kit (Click-iT EdU, Molecular Probes), according to the manufacturer's protocol, and stained with Hoechst 33342. The total DNA content was calculated by quantifying the level of Hoechst fluorescence using OpenLab, and a profile was constructed from over 700 cells.

### Comet assays

Comet assays were performed using a Trevigen Comet Assay Kit (4250-050-K). Briefly, cells were either untreated or treated with 100 µM Hydrogen Peroxide for 20 minutes at 4°C. Cells (∼1000) were mixed with low melt agarose, spotted onto slides, lysed, and electrophoresed under denaturing conditions at 4°C. DNA was stained with SYBR green and fluorescence was quantified using OpenLab. Damaged DNA is represented by the amount of signal present in the ‘tail’ as a percentage of the total. At least 50 cells were analyzed for each condition.

### DNA and RNA analysis

Genomic DNA for genotype analysis was purified from ear punch (at P21) and genotype was determined by PCR, as previously described. RNA was isolated and purified using Absolutely RNA kit (Stratagene). cDNA was generated using Superscript III (Invitrogen), and analyzed in triplicate by real time PCR using a BioRad MyIQ cycler and Sensimix Plus SYBRgreen plus FITC mix (Bioline), with intron spanning primer pairs (Table S8), selected using Primer3 (http://frodo.wi.mit.edu/). Expression was normalized to Rpl4 and Actin using the delta Ct method, and is shown as mean plus standard deviation of triplicates.

### Microarray Analysis

Biological triplicates of WT passage 3 and 5, and *Tgif1* null passage 3 MEFs were analyzed on Affymetrix MOE430_2.0 arrays. Data was normalized using the Bioconductor GCRMA algorithm. Microarray data was analyzed in compliance with the MIAME guidelines, and is deposited in the GEO database (GSE24225). Three pair-wise comparisons were performed and probe-sets selected at cutoff of 0.5 fold log base 2 change in expression, and a p-value of less than 0.0001. For comparisons with publicly available data-sets the p-value cutoff was altered to 0.001. 10 hour TGFβ treatment data was from GSE15871, and 4 hour TNFα treatment data was from GSE3700. Overlaps between data sets were generated using a Venn diagram generator (http://www.pangloss.com/seidel/Protocols/venn.cgi) Comparison of overlaps was performed using a 2×2 contingency table to calculate the expected distribution of the probe-set changes within the overlap between two data sets, followed by significance testing by Chi squared analysis. Pathway analysis and GO term assignation was performed using the DAVID functional annotation clustering tool (http://david.abcc.ncifcrf.gov) [37], [38].

### Western blotting and cell fractionation

Proteins were separated by SDS-PAGE, transferred to Immobilon-P (Millipore) and proteins were visualized using ECL (Pierce). Primary antibodies were against α-tubulin (Sigma), p19 (Abcam), p27 (BD Biosciences), Pten (Cell Signaling) Smad2/3 (Millipore), Smad4 (Millipore), phospho-Smad2 (Chemicon), CtBP1 (BD Biosciences) and Tgif1 [22], and were detected with a goat anti-rabbit secondary (Pierce). Digitonin and NP40 soluble fractions representing soluble cytosolic and nuclear proteins were isolated as described [49]. Wild type and *Tgif1* null fractions were run in parallel, transferred to a single membrane and probed together. For quantification of p19 and p27, membranes were incubated with IRDye (Li-Cor) goat anti mouse or rabbit secondary antibodies and scanned on an Odyssey Infrared Imager.

## Supporting Information

Table S1 **Gene expression array data.** WT passage 3 and 5, and *Tgif1* null passage 3 MEFs were analyzed on Affymetrix MOE430_2.0 arrays. All probe-sets making a 0.0001 p-value and 0.5 log-fold change cut-off are listed.
(XLS)

Table S2 **Tgif1-dependent gene expression changes.** All genes represented by probe-sets that showed at least a 2-fold change between wild type and *Tgif1* null P3 MEFs are listed.
(XLS)

Table S3 **GO term analysis of probe-sets with differential signal between** ***Tgif1*** **null and wild type P3 MEFs.** The top five clusters (both increased and decreased) generated by DAVID functional annotation clustering tool (http://david.abcc.ncifcrf.gov) are shown.
(DOC)

Table S4 **GO term analysis of probe-sets with differential signal between P3** ***Tgif1*** **null and wild type MEFs at both P3 and P5.** The top five clusters (increased) and top three clusters (decreased – clusters with an enrichment score below 1.5 were not included) generated by DAVID functional annotation clustering tool (http://david.abcc.ncifcrf.gov) are shown.
(DOC)

Table S5 **GO term analysis of probe-sets with differential signal between** ***Tgif1*** **null and wild type P3 MEFs and between wild type P5 and P3 MEFs.** The top five clusters (both increased and decreased) generated by DAVID functional annotation clustering tool (http://david.abcc.ncifcrf.gov) are shown.
(DOC)

Table S6 **GO term analysis of probe-sets with increased or decreased signal in comparisons between both P3** ***Tgif1*** **null and wild type MEFs (this work) and wild type MEFs treated with TGFβ (from GSE15871).** The top clusters with an enrichment score above 1.5, generated by DAVID functional annotation clustering tool (http://david.abcc.ncifcrf.gov) are shown.
(DOC)

Table S7 **GO term analysis of probe-sets with increased or decreased signal in comparisons between both P3 and P5 wild type MEFs (this work) and wild type MEFs treated with TGFβ (from GSE15871).** The top clusters (with a cutoff of an enrichment score >1.5 and p-values of the top GO terms<0.05) generated by DAVID functional annotation clustering tool (http://david.abcc.ncifcrf.gov) are shown.
(DOC)

Table S8 **Primers sets for qRT-PCR.** The sequences of forward and reverse primers (selected using Primer3 [http://frodo.wi.mit.edu/]) used for qRT-PCR are shown.
(DOC)

## References

[1] Feng XH, Derynck R (2005). Specificity and versatility in tgf-beta signaling through Smads.. Annu Rev Cell Dev Biol.

[2] Massague J, Seoane J, Wotton D (2005). Smad transcription factors.. Genes Dev.

[3] Schmierer B, Hill CS (2007). TGFbeta-SMAD signal transduction: molecular specificity and functional flexibility.. Nat Rev Mol Cell Biol.

[4] Alexandrow MG, Moses HL (1995). Transforming growth factor ß and cell cycle regulation.. Cancer Res.

[5] Datto MB, Li Y, Panus JF, Howe DJ, Xiong Y (1995). Transforming growth factor ß induces the cyclin-dependent kinase inhibitor p21 through a p53-independent mechanisms.. Proc Natl Acad Sci USA.

[6] Hannon GJ, Beach D (1994). p15INK4B is a potential effector of TGF-β-induced cell cycle arrest.. Nature.

[7] Warner BJ, Blain SW, Seoane J, Massague J (1999). Myc downregulation by transforming growth factor beta required for activation of the p15(Ink4b) G(1) arrest pathway.. Mol Cell Biol.

[8] Ikushima H, Miyazono K (2010). TGFbeta signalling: a complex web in cancer progression.. Nat Rev Cancer.

[9] Massague J, Blain SW, Lo RS (2000). TGFbeta signaling in growth control, cancer, and heritable disorders.. Cell.

[10] Bertolino E, Reimund B, Wildt-Perinic D, Clerc R (1995). A novel homeobox protein which recognizes a TGT core and functionally interferes with a retinoid-responsive motif.. J Biol Chem.

[11] Burglin TR (1997). Analysis of TALE superclass homeobox genes (MEIS, PBC, KNOX, Iroquois, TGIF) reveals a novel domain conserved between plants and animals.. Nucl Acids Res.

[12] Hyman CA, Bartholin L, Newfeld SJ, Wotton D (2003). Drosophila TGIF proteins are transcriptional activators.. Mol Cell Biol.

[13] Gripp KW, Wotton D, Edwards MC, Roessler E, Ades L (2000). Mutations in TGIF cause holoprosencephaly and link NODAL signalling to human neural axis determination.. Nat Genet.

[14] Muenke M, Beachy PA (2000). Genetics of ventral forebrain development and holoprosencephaly.. Curr Opin Genet Dev.

[15] Bartholin L, Powers SE, Melhuish TA, Lasse S, Weinstein M (2006). TGIF inhibits retinoid signaling.. Mol Cell Biol.

[16] Jin JZ, Gu S, McKinney P, Ding J (2006). Expression and functional analysis of Tgif during mouse midline development.. Dev Dyn.

[17] Mar L, Hoodless PA (2006). Embryonic fibroblasts from mice lacking Tgif were defective in cell cycling.. Mol Cell Biol.

[18] Shen J, Walsh CA (2005). Targeted disruption of Tgif, the mouse ortholog of a human holoprosencephaly gene, does not result in holoprosencephaly in mice.. Mol Cell Biol.

[19] Bartholin L, Melhuish TA, Powers SE, Goddard-Leon S, Treilleux I (2008). Maternal Tgif is required for vascularization of the embryonic placenta.. Dev Biol.

[20] Powers SE, Taniguchi K, Yen W, Melhuish TA, Shen J (2010). Tgif1 and Tgif2 regulate Nodal signaling and are required for gastrulation.. Development.

[21] Melhuish TA, Gallo CM, Wotton D (2001). TGIF2 interacts with histone deacetylase 1 and represses transcription.. J Biol Chem.

[22] Wotton D, Lo RS, Lee S, Massague J (1999). A Smad transcriptional corepressor.. Cell.

[23] Melhuish TA, Wotton D (2006). The Tgif2 gene contains a retained intron within the coding sequence.. BMC Mol Biol.

[24] Wotton D, Knoepfler PS, Laherty CD, Eisenman RN, Massague J (2001). The Smad Transcriptional Corepressor TGIF Recruits mSin3.. Cell Growth Differ.

[25] Melhuish TA, Wotton D (2000). The interaction of C-terminal binding protein with the Smad corepressor TG-interacting factor is disrupted by a holoprosencephaly mutation in TGIF.. J Biol Chem.

[26] Melhuish TA, Chung DD, Bjerke GA, Wotton D (2010). Tgif1 represses apolipoprotein gene expression in liver.. J Cell Biochem.

[27] Lombard DB, Chua KF, Mostoslavsky R, Franco S, Gostissa M (2005). DNA repair, genome stability, and aging.. Cell.

[28] Sherr CJ, DePinho RA (2000). Cellular senescence: mitotic clock or culture shock?. Cell.

[29] MacLaren A, Black EJ, Clark W, Gillespie DA (2004). c-Jun-deficient cells undergo premature senescence as a result of spontaneous DNA damage accumulation.. Mol Cell Biol.

[30] Mostoslavsky R, Chua KF, Lombard DB, Pang WW, Fischer MR (2006). Genomic instability and aging-like phenotype in the absence of mammalian SIRT6.. Cell.

[31] Borel F, Lohez OD, Lacroix FB, Margolis RL (2002). Multiple centrosomes arise from tetraploidy checkpoint failure and mitotic centrosome clusters in p53 and RB pocket protein-compromised cells.. Proc Natl Acad Sci U S A.

[32] Lanni JS, Lowe SW, Licitra EJ, Liu JO, Jacks T (1997). p53-independent apoptosis induced by paclitaxel through an indirect mechanism.. Proc Natl Acad Sci U S A.

[33] Sage J, Mulligan GJ, Attardi LD, Miller A, Chen S (2000). Targeted disruption of the three Rb-related genes leads to loss of G(1) control and immortalization.. Genes Dev.

[34] Uetake Y, Sluder G (2004). Cell cycle progression after cleavage failure: mammalian somatic cells do not possess a “tetraploidy checkpoint”.. J Cell Biol.

[35] Huang H, Fletcher L, Beeharry N, Daniel R, Kao G (2008). Abnormal cytokinesis after X-irradiation in tumor cells that override the G2 DNA damage checkpoint.. Cancer Res.

[36] Suzuki M, Suzuki K, Kodama S, Watanabe M (2006). Phosphorylated histone H2AX foci persist on rejoined mitotic chromosomes in normal human diploid cells exposed to ionizing radiation.. Radiat Res.

[37] Huang da W, Sherman BT, Lempicki RA (2009). Systematic and integrative analysis of large gene lists using DAVID bioinformatics resources.. Nat Protoc.

[38] Huang da W, Sherman BT, Lempicki RA (2009). Bioinformatics enrichment tools: paths toward the comprehensive functional analysis of large gene lists.. Nucleic Acids Res.

[39] Inman GJ, Nicolas FJ, Callahan JF, Harling JD, Gaster LM (2002). SB-431542 is a potent and specific inhibitor of transforming growth factor-beta superfamily type I activin receptor-like kinase (ALK) receptors ALK4, ALK5, and ALK7.. Mol Pharmacol.

[40] Wotton D, Massague J (2001). Smad transcriptional corepressors in TGF beta family signaling.. Curr Top Microbiol Immunol.

[41] Seo SR, Ferrand N, Faresse N, Prunier C, Abecassis L (2006). Nuclear retention of the tumor suppressor cPML by the homeodomain protein TGIF restricts TGF-beta signaling.. Mol Cell.

[42] Seo SR, Lallemand F, Ferrand N, Pessah M, L'Hoste S (2004). The novel E3 ubiquitin ligase Tiul1 associates with TGIF to target Smad2 for degradation.. Embo J.

[43] Ahmed SA, Gogal RMJ, Walsh JE (1994). A new rapid and simple non-radioactive assay to monitor and determine the proliferation of lymphocytes: an alternative to [3H]thymidine incorporation assay.. J Immunol Methods.

[44] Bartholin L, Maguer-Satta V, Hayette S, Martel S, Gadoux M (2002). Transcription activation of FLRG and follistatin by activin A, through Smad proteins, participates in a negative feedback loop to modulate activin A function.. Oncogene.

[45] Lo RS, Wotton D, Massague J (2001). Epidermal growth factor signaling via Ras controls the Smad transcriptional co-repressor TGIF.. Embo J.

[46] Hamid R, Brandt SJ (2009). Transforming growth-interacting factor (TGIF) regulates proliferation and differentiation of human myeloid leukemia cells.. Mol Oncol.

[47] Rodier F, Campisi J (2011). Four faces of cellular senescence.. J Cell Biol.

[48] Taniguchi K, Anderson AE, Sutherland AE, Wotton D (2012). Loss of Tgif function causes holoprosencephaly by disrupting the Shh signaling pathway.. PLoS Genet.

[49] Merrill JC, Kagey MH, Melhuish TA, Powers SE, Zerlanko BJ (2010). Inhibition of CtBP1 activity by Akt-mediated phosphorylation.. J Mol Biol.

