# Supplementary material for: Premature Senescence and Increased TGFβ Signaling in the Absence of Tgif1
Source: PLoS One. 2012 Apr 13;7(4):e35460. doi: 10.1371/journal.pone.0035460 (PMC3325954; doi:10.1371/journal.pone.0035460)
Supplement: Table S3 — GO term analysis of probe-sets with differential signal between Tgif1 null and wild type P3 MEFs. The top five clusters (both increased and decreased) generated by DAVID functional annotation clustering tool (http://david.abcc.ncifcrf.gov) are shown. (DOC) [file pone.0035460.s003.doc]

**Table S3. GO term analysis of probe-sets with differential signal between *Tgif1* null and wild type P3 MEFs.**

| **Change^1^** | **Cluster^2^** | **Score^3^** | **Term^4^** | **p value** |
| --- | --- | --- | --- | --- |
| Increased | 1 | 4.60 | GO:0043292 contractile fiber | 0.000002 |
|  |  |  | GO:0044449 contractile fiber part | 0.000005 |
|  |  |  | GO:0030016 myofibril | 0.000009 |
|  |  |  | GO:0030017 sarcomere | 0.000017 |
|  |  |  | GO:0031674 I band | 0.000226 |
|  | 2 | 4.06 | GO:0007155 cell adhesion | 0.000001 |
|  |  |  | GO:0022610 biological adhesion | 0.000001 |
|  |  |  | GO:0016337 cell-cell adhesion | 0.007478 |
|  | 3 | 3.95 | GO:0008219 cell death | 0.000033 |
|  |  |  | GO:0016265 death | 0.000050 |
|  |  |  | GO:0006915 apoptosis | 0.000147 |
|  |  |  | GO:0012501 programmed cell death | 0.000191 |
|  | 4 | 3.12 | GO:0042692 muscle cell differentiation | 0.000013 |
|  |  |  | GO:0014706 striated muscle tissue development | 0.000142 |
|  |  |  | GO:0051146 striated muscle cell differentiation | 0.000178 |
|  |  |  | GO:0007517 muscle organ development | 0.000182 |
|  |  |  | GO:0055002 striated muscle cell development | 0.000200 |
|  | 5 | 3.01 | GO:0043292 contractile fiber | 0.000002 |
|  |  |  | GO:0044449 contractile fiber part | 0.000005 |
|  |  |  | GO:0005200 structural constituent of cytoskeleton | 0.026761 |
|  |  |  | GO:0006936 muscle contraction | 0.046447 |
|  |  |  | GO:0003012 muscle system process | 0.064745 |
| Decreased | 1 | 10.46 | GO:0051301 cell division | 1.40E-14 |
|  |  |  | GO:0007067 mitosis | 2.06E-14 |
|  |  |  | GO:0000280 nuclear division | 2.06E-14 |
|  |  |  | GO:0000087 M phase of mitotic cell cycle | 3.35E-14 |
|  |  |  | GO:0048285 organelle fission | 4.79E-14 |
|  | 2 | 9.58 | GO:0006260 DNA replication | 7.68E-11 |
|  |  |  | GO:0006259 DNA metabolic process | 1.89E-09 |
|  | 3 | 3.51 | GO:0043232 intracellular non-membrane-bounded organelle | 5.86E-07 |
|  |  |  | GO:0043228 non-membrane-bounded organelle | 5.86E-07 |
|  |  |  | GO:0015630 microtubule cytoskeleton | 0.000043 |
|  |  |  | GO:0044430 cytoskeletal part | 0.001051 |
|  |  |  | GO:0005815 microtubule organizing center | 0.001826 |
|  | 4 | 3.37 | GO:0000902 cell morphogenesis | 0.000025 |
|  |  |  | GO:0048812 neuron projection morphogenesis | 0.000031 |
|  |  |  | GO:0048858 cell projection morphogenesis | 0.000032 |
|  |  |  | GO:0048667 cell morphogenesis involved in neuron differentiation | 0.000044 |
|  |  |  | GO:0032990 cell part morphogenesis | 0.000054 |
|  | 5 | 3.20 | GO:0035295 tube development | 0.000161 |
|  |  |  | GO:0060541 respiratory system development | 0.000593 |
|  |  |  | GO:0030324 lung development | 0.001242 |
|  |  |  | GO:0030323 respiratory tube development | 0.001394 |

Footnotes:

1. Increased or decreased signal in *Tgif1* null compared to wild type P3 MEFs.

2. The top five clusters (both increased and decreased) generated by DAVID functional annotation clustering tool ([http://david.abcc.ncifcrf.gov](http://david.abcc.ncifcrf.gov/)) are shown. The five GO terms with the best p values are shown for clusters with more than five terms.

3. The enrichment score is shown for each cluster.

4. GO terms within each cluster are listed.
