# Supplementary material for: Premature Senescence and Increased TGFβ Signaling in the Absence of Tgif1
Source: PLoS One. 2012 Apr 13;7(4):e35460. doi: 10.1371/journal.pone.0035460 (PMC3325954; doi:10.1371/journal.pone.0035460)
Supplement: Table S4 — GO term analysis of probe-sets with differential signal between P3 Tgif1 null and wild type MEFs at both P3 and P5. The top five clusters (increased) and top three clusters (decreased – clusters with an enrichment score below 1.5 were not included) generated by DAVID functional annotation clustering tool (http://david.abcc.ncifcrf.gov) are shown. (DOC) [file pone.0035460.s004.doc]

**Table S4. GO term analysis of probe-sets with differential signal between P3 *Tgif1* null and wild type MEFs at both P3 and P5.**

| **Change^1^** | **Cluster^2^** | **Score^3^** | **Term^4^** | **p value** |
| --- | --- | --- | --- | --- |
| Increased | 1 | 3.02 | GO:0015629 actin cytoskeleton | 0.000003 |
|  |  |  | GO:0005856 cytoskeleton | 0.000083 |
|  |  |  | GO:0044430 cytoskeletal part | 0.000112 |
|  |  |  | GO:0043228 non-membrane-bounded organelle | 0.045215 |
|  |  |  | GO:0043232 intracellular non-membrane-bounded organelle | 0.045215 |
|  | 2 | 2.70 | GO:0007155 cell adhesion | 0.000507 |
|  |  |  | GO:0022610 biological adhesion | 0.000517 |
|  | 3 | 2.47 | GO:0015629 actin cytoskeleton | 0.000003 |
|  |  |  | GO:0003774 motor activity | 0.000017 |
|  |  |  | GO:0016459 myosin complex | 0.000055 |
|  |  |  | GO:0044430 cytoskeletal part | 0.000112 |
|  |  |  | GO:0005516 calmodulin binding | 0.122814 |
|  | 4 | 2.45 | GO:0008092 cytoskeletal protein binding | 0.001614 |
|  |  |  | GO:0051015 actin filament binding | 0.001824 |
|  |  |  | GO:0003779 actin binding | 0.003776 |
|  | 5 | 3.01 | GO:0048514 blood vessel morphogenesis | 0.001660 |
|  |  |  | GO:0001568 blood vessel development | 0.005929 |
|  |  |  | GO:0001944 vasculature development | 0.006832 |
|  |  |  | GO:0001525 angiogenesis | 0.016377 |
| Decreased | 1 | 2.48 | GO:0048812 neuron projection morphogenesis | 0.000160 |
|  |  |  | GO:0048858 cell projection morphogenesis | 0.000336 |
|  |  |  | GO:0048666 neuron development | 0.000389 |
|  |  |  | GO:0032990 cell part morphogenesis | 0.000435 |
|  |  |  | GO:0030182 neuron differentiation | 0.000486 |
|  | 2 | 1.82 | GO:0001568 blood vessel development | 0.005402 |
|  |  |  | GO:0001944 vasculature development | 0.005978 |
|  |  |  | GO:0048514 blood vessel morphogenesis | 0.013347 |
|  |  |  | GO:0001525 angiogenesis | 0.126474 |
|  | 3 | 1.55 | GO:0019210 kinase inhibitor activity | 0.004659 |
|  |  |  | GO:0019207 kinase regulator activity | 0.045557 |
|  |  |  | GO:0004857 enzyme inhibitor activity | 0.104389 |

Footnotes:

1. Increased or decreased signal in P3 *Tgif1* null MEFs compared to wild type P3 and wild type P5 MEFs.

2. The top five clusters (increased) and top three clusters (decreased – clusters with an enrichment score below 1.5 were not included) generated by DAVID functional annotation clustering tool ([http://david.abcc.ncifcrf.gov](http://david.abcc.ncifcrf.gov/)) are shown. The five GO terms with the best p values are shown for clusters with more than five terms.

3. The enrichment score is shown for each cluster.

4. GO terms within each cluster are listed.
