# Supplementary material for: Premature Senescence and Increased TGFβ Signaling in the Absence of Tgif1
Source: PLoS One. 2012 Apr 13;7(4):e35460. doi: 10.1371/journal.pone.0035460 (PMC3325954; doi:10.1371/journal.pone.0035460)
Supplement: Table S5 — GO term analysis of probe-sets with differential signal between Tgif1 null and wild type P3 MEFs and between wild type P5 and P3 MEFs. The top five clusters (both increased and decreased) generated by DAVID functional annotation clustering tool (http://david.abcc.ncifcrf.gov) are shown. (DOC) [file pone.0035460.s005.doc]

**Table S5. GO term analysis of probe-sets with differential signal between *Tgif1* null and wild type P3 MEFs and between wild type P5 and P3 MEFs.**

| **Change^1^** | **Cluster^2^** | **Score^3^** | **Term^4^** | **p value** |
| --- | --- | --- | --- | --- |
| Increased | 1 | 2.64 | GO:0042981 regulation of apoptosis | 0.000864 |
|  |  |  | GO:0043067 regulation of programmed cell death | 0.000970 |
|  |  |  | GO:0010941 regulation of cell death | 0.001018 |
|  |  |  | GO:0008219 cell death | 0.004028 |
|  |  |  | GO:0016265 death | 0.004796 |
|  | 2 | 2.60 | GO:0042981 regulation of apoptosis | 0.000864 |
|  |  |  | GO:0043067 regulation of programmed cell death | 0.000970 |
|  |  |  | GO:0010941 regulation of cell death | 0.001018 |
|  |  |  | GO:0043066 negative regulation of apoptosis | 0.004390 |
|  |  |  | GO:0043069 negative regulation of programmed cell death | 0.004910 |
|  | 3 | 2.36 | GO:0043167 ion binding | 0.000291 |
|  |  |  | GO:0046872 metal ion binding | 0.000327 |
|  |  |  | GO:0043169 cation binding | 0.000416 |
|  |  |  | GO:0046914 transition metal ion binding | 0.017716 |
|  |  |  | GO:0008270 zinc ion binding | 0.028098 |
|  | 4 | 2.32 | GO:0044449 contractile fiber part | 0.001117 |
|  |  |  | GO:0043292 contractile fiber | 0.001741 |
|  |  |  | GO:0030017 sarcomere | 0.006089 |
|  |  |  | GO:0030018 Z disc | 0.008427 |
|  |  |  | GO:0030016 myofibril | 0.009536 |
|  | 5 | 2.24 | GO:0007155 cell adhesion | 0.000986 |
|  |  |  | GO:0022610 biological adhesion | 0.001002 |
|  |  |  | GO:0016337 cell-cell adhesion | 0.051626 |
| Decreased | 1 | 13.09 | GO:0006259 DNA metabolic process | 1.20E-14 |
|  |  |  | GO:0006260 DNA replication | 4.20E-14 |
|  | 2 | 12.84 | GO:0051301 cell division | 1.66E-17 |
|  |  |  | GO:0007049 cell cycle | 1.74E-17 |
|  |  |  | GO:0000279 M phase | 2.70E-16 |
|  |  |  | GO:0022403 cell cycle phase | 4.97E-16 |
|  |  |  | GO:0022402 cell cycle process | 2.57E-15 |
|  | 3 | 6.48 | GO:0005694 chromosome | 6.78E-13 |
|  |  |  | GO:0044427 chromosomal part | 5.00E-12 |
|  |  |  | GO:0043232 intracellular non-membrane-bounded organelle | 7.80E-12 |
|  |  |  | GO:0043228 non-membrane-bounded organelle | 7.80E-12 |
|  |  |  | GO:0044430 cytoskeletal part | 0.000054 |
|  | 4 | 3.45 | GO:0003887 DNA-directed DNA polymerase activity | 0.001175 |
|  |  |  | GO:0034061 DNA polymerase activity | 0.004571 |
|  |  |  | GO:0016779 nucleotidyltransferase activity | 0.059528 |
|  | 5 | 2.71 | GO:0006281 DNA repair | 0.000342 |
|  |  |  | GO:0006974 response to DNA damage stimulus | 0.000405 |
|  |  |  | GO:0006310 DNA recombination | 0.002781 |
|  |  |  | GO:0033554 cellular response to stress | 0.004300 |

Footnotes:

1. Increased or decreased signal in both *Tgif1* null compared to wild type P3 MEFs and in wild type P5 compared to wild type P3 MEFs.

2. The top five clusters (both increased and decreased) generated by DAVID functional annotation clustering tool ([http://david.abcc.ncifcrf.gov](http://david.abcc.ncifcrf.gov/)) are shown. The five GO terms with the best p values are shown for clusters with more than five terms.

3. The enrichment score is shown for each cluster.

4. GO terms within each cluster are listed.
