# Supplementary material for: Premature Senescence and Increased TGFβ Signaling in the Absence of Tgif1
Source: PLoS One. 2012 Apr 13;7(4):e35460. doi: 10.1371/journal.pone.0035460 (PMC3325954; doi:10.1371/journal.pone.0035460)
Supplement: Table S6 — GO term analysis of probe-sets with increased or decreased signal in comparisons between both P3 Tgif1 null and wild type MEFs (this work) and wild type MEFs treated with TGFβ (from GSE15871). The top clusters with an enrichment score above 1.5, generated by DAVID functional annotation clustering tool (http://david.abcc.ncifcrf.gov) are shown. (DOC) [file pone.0035460.s006.doc]

**Table S6. GO term analysis of probe-sets with increased or decreased signal in comparisons between both P3 *Tgif1* null and wild type MEFs (our data) and wild type MEFs treated with TGFβ (from GSE15871).**

| **Change^1^** | **Cluster^2^** | **Score^3^** | **Term^4^** | **p value** |
| --- | --- | --- | --- | --- |
| Increased | 1 | 1.57 | GO:0001525 angiogenesis | 0.036749 |
|  |  |  | GO:0048514 blood vessel morphogenesis | 0.074578 |
|  |  |  | GO:0001568 blood vessel development | 0.106379 |
|  |  |  | GO:0001944 vasculature development | 0.110767 |
| Decreased | 1 | 4.66 | GO:0007049 cell cycle | 5.67E-08 |
|  |  |  | GO:0051301 cell division | 5.87E-08 |
|  |  |  | GO:0022402 cell cycle process | 8.38E-08 |
|  |  |  | GO:0000280 nuclear division | 8.18E-07 |
|  |  |  | GO:0007067 mitosis | 8.18E-07 |
|  | 2 | 2.23 | GO:0006260 DNA replication | 0.000004 |
|  |  |  | GO:0006259 DNA metabolic process | 0.000143 |
|  |  |  | GO:0003677 DNA binding | 0.039580 |
|  |  |  | GO:0006350 transcription | 0.889080 |
|  | 3 | 2.21 | GO:0043228 non-membrane-bounded organelle | 0.000366 |
|  |  |  | GO:0043232 intracellular non-membrane-bounded organelle | 0.000366 |
|  |  |  | GO:0015630 microtubule cytoskeleton | 0.001404 |
|  |  |  | GO:0005856 cytoskeleton | 0.003022 |
|  |  |  | GO:0044430 cytoskeletal part | 0.004962 |

Footnotes:

1. Increased or decreased signal in both *Tgif1* null MEFs compared to wild type P3 and in wild type MEFs treated with TGFβ.

2. The top clusters with an enrichment score above 1.5, generated by DAVID functional annotation clustering tool ([http://david.abcc.ncifcrf.gov](http://david.abcc.ncifcrf.gov/)) are shown. The five GO terms with the best p values are shown for clusters with more than five terms.

3. The enrichment score is shown for each cluster.

4. GO terms within each cluster are listed.
