# Supplementary material for: Premature Senescence and Increased TGFβ Signaling in the Absence of Tgif1
Source: PLoS One. 2012 Apr 13;7(4):e35460. doi: 10.1371/journal.pone.0035460 (PMC3325954; doi:10.1371/journal.pone.0035460)
Supplement: Table S7 — GO term analysis of probe-sets with increased or decreased signal in comparisons between both P3 and P5 wild type MEFs (this work) and wild type MEFs treated with TGFβ (from GSE15871). The top clusters (with a cutoff of an enrichment score >1.5 and p-values of the top GO terms<0.05) generated by DAVID functional annotation clustering tool (http://david.abcc.ncifcrf.gov) are shown. (DOC) [file pone.0035460.s007.doc]

**Table S7. GO term analysis of probe-sets with increased or decreased signal in comparisons between both P3 and P5 wild type MEFs (our data) and wild type MEFs treated with TGFβ (from GSE15871).**

| **Change^1^** | **Cluster^2^** | **Score^3^** | **Term^4^** | **p value** |
| --- | --- | --- | --- | --- |
| Increased | 1 | 3.27 | GO:0051094 positive regulation of developmental process | 1.45E-07 |
|  |  |  | GO:0045597 positive regulation of cell differentiation | 0.000006 |
|  |  |  | GO:0008284 positive regulation of cell proliferation | 0.000880 |
|  |  |  | GO:0051240 positive regulation of multicellular organismal process | 0.026910 |
|  |  |  | GO:0009967 positive regulation of signal transduction | 0.030874 |
|  | 2 | 3.12 | GO:0045597 positive regulation of cell differentiation | 0.000006 |
|  |  |  | GO:0045639 positive regulation of myeloid cell differentiation | 0.004386 |
|  |  |  | GO:0045637 regulation of myeloid cell differentiation | 0.017453 |
|  | 3 | 2.65 | GO:0045597 positive regulation of cell differentiation | 0.000006 |
|  |  |  | GO:0048732 gland development | 0.043408 |
|  |  |  | GO:0030879 mammary gland development | 0.045827 |
|  | 4 | 1.98 | GO:0008284 positive regulation of cell proliferation | 0.000880 |
|  |  |  | GO:0001568 blood vessel development | 0.002768 |
|  |  |  | GO:0001944 vasculature development | 0.003073 |
|  |  |  | GO:0048514 blood vessel morphogenesis | 0.007838 |
|  |  |  | GO:0001570 vasculogenesis | 0.012613 |
| Decreased | 1 | 20.67 | GO:0000279 M phase | 9.31E-24 |
|  |  |  | GO:0007049 cell cycle | 1.40E-23 |
|  |  |  | GO:0022403 cell cycle phase | 1.64E-23 |
|  |  |  | GO:0022402 cell cycle process | 8.69E-23 |
|  |  |  | GO:0000278 mitotic cell cycle | 1.75E-22 |
|  | 2 | 14.58 | GO:0044427 chromosomal part | 5.53E-23 |
|  |  |  | GO:0005694 chromosome | 1.46E-22 |
|  |  |  | GO:0000775 chromosome, centromeric region | 3.10E-18 |
|  |  |  | GO:0043228 non-membrane-bounded organelle | 7.83E-18 |
|  |  |  | GO:0043232 intracellular non-membrane-bounded organelle | 7.83E-18 |
|  | 3 | 9.56 | GO:0043228 non-membrane-bounded organelle | 7.83E-18 |
|  |  |  | GO:0043232 intracellular non-membrane-bounded organelle | 7.83E-18 |
|  |  |  | GO:0015630 microtubule cytoskeleton | 2.30E-12 |
|  |  |  | GO:0044430 cytoskeletal part | 2.22E-09 |
|  |  |  | GO:0005819 spindle | 2.47E-09 |
|  | 4 | 4.62 | GO:0006260 DNA replication | 4.16E-15 |
|  |  |  | GO:0006259 DNA metabolic process | 1.08E-12 |
|  |  |  | GO:0003887 DNA-directed DNA polymerase activity | 0.000432 |
|  |  |  | GO:0006281 DNA repair | 0.001500 |
|  |  |  | GO:0034061 DNA polymerase activity | 0.001726 |
|  | 5 | 4.36 | GO:0005819 spindle | 2.47E-09 |
|  |  |  | GO:0005874 microtubule | 0.000002 |
|  |  |  | GO:0015631 tubulin binding | 0.000036 |
|  |  |  | GO:0005876 spindle microtubule | 0.000072 |
|  |  |  | GO:0008017 microtubule binding | 0.003430 |

Footnotes:

1. Increased or decreased signal in both P3 compared to P5 wild type MEFs and in wild type MEFs treated with TGFβ.

2. The top clusters (with a cutoff of an enrichment score > 1.5 and p-values of the top GO terms < 0.05) generated by DAVID functional annotation clustering tool ([http://david.abcc.ncifcrf.gov](http://david.abcc.ncifcrf.gov/)) are shown. The five GO terms with the best p values are shown for clusters with more than five terms.

3. The enrichment score is shown for each cluster.

4. GO terms within each cluster are listed.
