# Supplementary material for: Premature Senescence and Increased TGFβ Signaling in the Absence of Tgif1
Source: PLoS One. 2012 Apr 13;7(4):e35460. doi: 10.1371/journal.pone.0035460 (PMC3325954; doi:10.1371/journal.pone.0035460)
Supplement: Table S8 — Primers sets for qRT-PCR. The sequences of forward and reverse primers (selected using Primer3 [http://frodo.wi.mit.edu/]) used for qRT-PCR are shown. (DOC) [file pone.0035460.s008.doc]

**Supplemental Table 8**

**Primers sets for qRT-PCR:**

**Gene F/R Sequence**

Bub1b F GGAAACTTAGCCAGAGCACCT

R AAAAGCCCCACCCATTTC

Olr1 F GTCATCCTCTGCCTGGTGTT

R GCTGAGTAAGGTTCGCTTGG

Leprel1 F CAAAGTGTGGCCGAATGA

R ATCTGCCTGTATCCGCTAGTTC

Loxl2 F CCCAACTATGAAGTGCCAGA

R GGCCCAGGATCTTCTTTACT

Fas F TCCTGCCTCTTGCTTGCT

R AGGTTGGCATGGTTGACAG

Enpep F CTGCTCTATGGGTTAGCTTCAG

R GTTGTAGGAGATGTAGCGGATG

Stmn4 F TTAATGCCTCCCTCCCAAG

R CTGGTTTCCTTCGCTCCTCT

Mgll F GTGCCTACCTGCTCATGGA

R GAGGACGGAGTTGGTCACTT

CD40 F GACAACACTGCACCAGCAA

R ATTGGAGAAGAAGCCGACTG

Gas1 F ACCCTGCAAAAGCAAAGG

R GGTTCCTGGTGAGCAATGTA

Mboat1 F GTCGAGCCAACCATCAGTTT

R CCGTTTCTCTGTGGGTATCAG

Rasl11b F GTGGTCCGATTCCTCACTAAAC

R TAGCCAGCGTTTCTCCTTCT

Rfc5 F GGGCTTTGAACATCCTTCAG

R TGATTGAGCATCCAGTCCAG

Cdc45l F GCTCAGGGACAGACCACTTC

R CGAGGTTGGTACAGAGACAGC

Pole F GGTTCACACTTGCTGCTCAA

R AACGTCTACCAGGCGAAGAA

Plk4 F TGGAAATGGTACAGCAGTGG

R ACGTGTCCTGCAAATCTGG

Cflar F TGCACAGCAGACGTATCTCA

R TCCACGCATACACTTTGTCC

Herpud1 F CAACCAGGACCCCAACAATA

R CCATGCTGTGCTCATAAACG

Cdc25b F AAAGGCGGCTACAAGGAGTT

R TAAGGCGAAAGTTCCTCAGC

Tgfb1 F ATGACATGAACCGGCCCTTCCT

R TGCCGCACACAGCAGTTCTTCTC

Tgfbr1 F AACCGCACTGTCATTCACCACCG

R TCGCCAAACTTCTCCAAACCGACC

Tgfbr2 F CTTGCGACAACCAGAAGTCC

R GGCATCTTCCAGAGTGAAGC

Smad7 F TCAAGAGGCTGTGTTGCTGT

R TGGGTATCTGGAGTAAGGAGGA

Skil F GCAGCAGAAATGCACCTGT

R CCGCTCCTGTCTGAGTTCAT
